# *Candida glabrata* maintains two *HAP1* ohnologs, *HAP1A* and *HAP1B*, for distinct roles in ergosterol gene regulation to mediate sterol homeostasis under azole and hypoxic conditions

**DOI:** 10.1128/msphere.00524-24

**Published:** 2024-10-23

**Authors:** Debasmita Saha, Justin B. Gregor, Smriti Hoda, Katharine E. Eastman, Victor A. Gutierrez-Schultz, Mindy Navarrete, Jennifer H. Wisecaver, Scott D. Briggs

**Affiliations:** 1 Department of Biochemistry, Purdue University, West Lafayette, Indiana, USA; 2 Purdue University Institute for Cancer Research, West Lafayette, Indiana, USA; University of Georgia, Athens, Georgia, USA

**Keywords:** *Candida glabrata*, zinc cluster transcription factors, azole antifungal drugs, drug resistance mechanisms, gene regulation, hypoxia, ergosterol pathway, *ERG11*, *ERG3*, Hap1, *HAP1A *(*ZCF4*,* MAR1*), *HAP1B *(*ZCF27*,* HAP1*)

## Abstract

*Candida glabrata* exhibits innate resistance to azole antifungal drugs but also has the propensity to rapidly develop clinical drug resistance. Azole drugs, which target Erg11, is one of the major classes of antifungals used to treat *Candida* infections. Despite their widespread use, the mechanism controlling azole-induced *ERG* gene expression and drug resistance in *C. glabrata* has primarily revolved around Upc2 and/or Pdr1. Phylogenetic and syntenic analyses revealed that *C. glabrata*, following a whole genome duplication event, maintained *HAP1A* and *HAP1B*, whereas *Saccharomyces cerevisiae* only retained the *HAP1A* ortholog, *HAP1*. In this study, we determined the function of two zinc cluster transcription factors, Hap1A and Hap1B, as direct regulators of *ERG* genes. In *S. cerevisiae,* Hap1, an ortholog of Hap1A, is a known transcription factor controlling *ERG* gene expression under aerobic and hypoxic conditions. Interestingly, deleting *HAP1* or *HAP1B* in either *S. cerevisiae* or *C. glabrata,* respectively, showed altered susceptibility to azoles. In contrast, the strain deleted for *HAP1A* did not exhibit azole susceptibility. We also determined that the increased azole susceptibility in a *hap1B*Δ strain is attributed to decreased azole-induced expression of *ERG* genes, resulting in decreased levels of total ergosterol. Surprisingly, Hap1A protein expression is barely detected under aerobic conditions but is specifically induced under hypoxic conditions, where Hap1A is required for the repression of *ERG* genes. However, in the absence of Hap1A, Hap1B can compensate as a transcriptional repressor. Our study shows that Hap1A and Hap1B is utilized by *C. glabrata* to adapt to specific host and environmental conditions.

**IMPORTANCE:**

Invasive and drug-resistant fungal infections pose a significant public health concern. *Candida glabrata*, a human fungal pathogen, is often difficult to treat due to its intrinsic resistance to azole antifungal drugs and its capacity to develop clinical drug resistance. Therefore, understanding the pathways that facilitate fungal growth and environmental adaptation may lead to novel drug targets and/or more efficacious antifungal therapies. While the mechanisms of azole resistance in *Candida* species have been extensively studied, the roles of zinc cluster transcription factors, such as Hap1A and Hap1B, in *C. glabrata* have remained largely unexplored until now. Our research shows that these factors play distinct yet crucial roles in regulating ergosterol homeostasis under azole drug treatment and oxygen-limiting growth conditions. These findings offer new insights into how this pathogen adapts to different environmental conditions and enhances our understanding of factors that alter drug susceptibility and/or resistance.

## INTRODUCTION

Invasive and drug-resistant fungal infections are significant public health issues, and new estimates indicate that life-threatening fungal infections affect over 6.5 million people globally each year (1). Among these global invasive fungal infections, more than 70% are caused by invasive *Candida* species, which include *Candida albicans* and other non-*albicans* (NAC) *Candida* species, such as *C. glabrata*, *C. krusei*, *C. tropicalis*, and *C. parapsilosis* (2–5). Of the NAC species listed, *Candida glabrata* is considered the second or third most commonly isolated NAC *Candid*a species, with *C. albicans* being the most commonly isolated (2, 4–6). The traditional genus *Candida* is a paraphyletic group, and *C. glabrata* is more closely related to *Saccharomyces cerevisiae* than to other common human pathogens, including *C. albicans* (7). The last common ancestor (LCA) of *C. glabrata* and *C. albicans* existed ~250 million years ago (Mya), whereas the LCA of *C. glabrata* and *S. cerevisiae* occurred ~50 Mya (8). *C. glabrata* is considered the major pathogenic species of the post-whole genome duplication (WGD) *Saccharomycetaceae* group, with immunosuppressed patients (e.g., those with diabetes mellitus, cancer, or organ transplants) and/or elderly patients being particularly susceptible to these infections (6, 9–12).

*C. glabrata* is also a non-CTG clade *Candida* species that is known for its intrinsic resistance to azole drugs and ability to develop clinical azole drug resistance (7, 13, 14). Azole drugs target and inhibit the enzyme lanosterol 14-α-demethylase (Erg11), which is an essential enzyme to produce ergosterol in fungi (15–17). Mechanisms of acquiring clinical azole drug resistance have been extensively documented across *Candida* species and include mutations in *ERG11*, *ERG3*, *UPC2*, and/or *PDR1* (14, 18–25). Among these genes, gain of function (GOF) mutations in the zinc cluster transcription factors Upc2 and Pdr1 result in increased expression of *ERG11* and/or the ABC drug transporter *CDR1,* respectively (24, 26–30). As for *C. glabrata* clinical drug-resistant isolates, Pdr1 GOF mutations are considered the predominant cause for clinical drug resistance (28, 31).

In addition to Upc2 and Pdr1, several known and/or putative zinc cluster factors (Zcfs) are critical transcriptional regulators involved in stress response in fungi and amoeba (32, 33). Interestingly, 17 of the 41 *C*. *glabrata ZCF* genes when deleted show enhanced azole susceptibility as indicated by MIC and/or plate-based growth assays (34). This observation underscores the importance and need to further investigate the role of these zinc cluster transcription factors in *C. glabrata*. However, with the exception of Upc2A (*Zcf5*), Pdr1 (*Zcf1*), Stb5 (*Zcf24*), and Hap1A (*Mar1*, *Zcf4*), little research has been done to understand the mechanistic role of other *C. glabrata* Zcf proteins during azole treatment conditions and/or hypoxic growth (30, 35–39).

In this report, we show for the first time that *S. cerevisiae* strains deleted for *HAP1* exhibit azole hypersusceptibility when compared to a FY2609 WT strain containing a WT copy of the *HAP1* gene. Interestingly, S288C strains, including the commonly used BY4741 and BY4742, exhibit similar azole susceptibility to *hap1*Δ strains due to a partially disrupted *hap1* gene by a *Ty1* element (*hap1-Ty1* mutation). From syntenic and phylogenetic analysis, *C. glabrata* contains two ohnologs (gene duplicates originating from whole genome duplication), Hap1A (Zcf4, Mar1) and Hap1B (Zcf27, Hap1), which are homologs of *S. cerevisiae* Hap1. Based on these observations, we hypothesized that deletion of *C. glabrata HAP1* ohnologs would also have a similar azole susceptible phenotype. However, only deletion of *C. glabrata HAP1B,* but not *HAP1A,* showed an azole hypersusceptible phenotype. Upon further investigation, we established that altered azole susceptibility of the *hap1B*Δ strain is attributed to a decrease in azole-induced *ERG* gene expression, resulting in a subsequent reduction in total ergosterol levels. Moreover, azole hypersusceptibility of the *hap1B*Δ strain was alleviated when complemented with a plasmid expressing *HAP1B* or when exogenous ergosterol was introduced into the growth media, but not when the *AUS1* sterol transporter was deleted. Interestingly, unlike Hap1B, Hap1A protein levels were nearly undetectable under both untreated and azole-treated conditions. However, under hypoxic conditions, Hap1A was highly induced, while the expression of Hap1B remained unchanged. Moreover, the *hap1A*Δ strain showed a growth defect that correlated with a failure to repress *ERG* genes under hypoxic conditions, while the *hap1B*Δ strain grew similar to *Cg*2001 WT and maintained *ERG* gene repression. Interestingly, the hypoxic growth defect and failure to repress *ERG* genes is further exacerbated in a *hap1A*Δ*hap1B*Δ double-deletion strain, suggesting Hap1B can compensate for the loss of Hap1A. Additionally, our studies demonstrated that Hap1B and Hap1A can associate with promoters of *ERG* genes, and their enrichment at these sites is further enhanced upon azole treatment or hypoxic conditions, respectively. Overall, we have discovered that *C. glabrata* maintains two Hap1 ohnologs to regulate ergosterol homeostasis. Specifically, Hap1B aids in facilitating azole-mediated gene activation, while Hap1A mediates hypoxia-induced gene repression.

## RESULTS

### Hap1 alters azole susceptibility in *S. cerevisiae*

**Fig 1:**
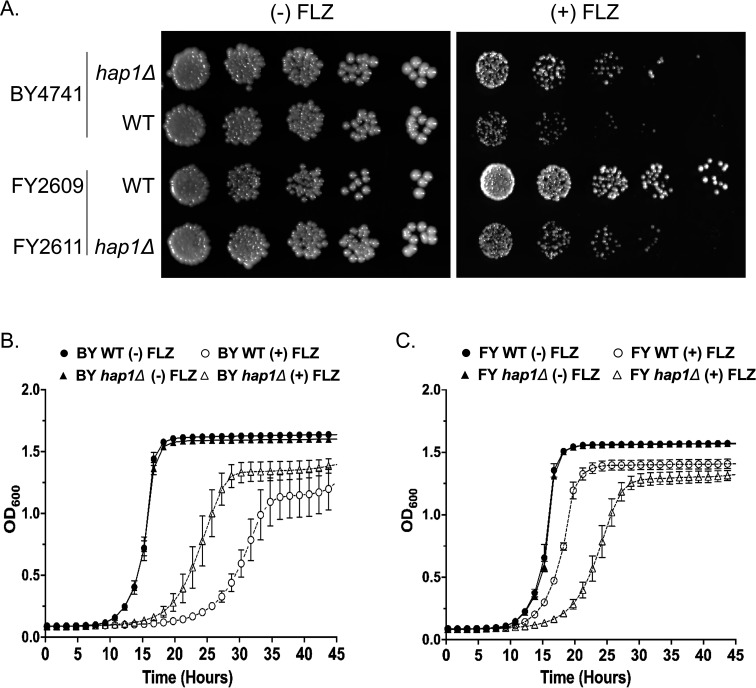
Zinc cluster transcription factor Hap1 in *S. cerevisiae* alters fluconazole susceptibility. (**A**) Fluconazole susceptibility of BY4741, BY4741 *hap1*Δ, FY2609, and FY2611 *hap1*Δ of *S. cerevisiae* S288C strains. Fivefold serial dilution assays of indicated strains grown on SC plates with and without 16 µg/mL fluconazole and incubated at 30°C for 48 h. A minimum of three biological replicates were performed. (**B and C**) Growth curve of indicated strains grown in SC liquid media with or without 16 µg/mL fluconazole. Error bars represent SD.

In *S. cerevisiae*, there are three zinc cluster transcription factors Upc2, Ecm22, and Hap1 that are known to regulate the expression of ergosterol gene expression for sterol homeostasis (40–44). In addition, Upc2 and Ecm22 are also known to mediate azole susceptibility in *S. cerevisiae* (45, 46). However, until now, the role of Hap1 in altering azole susceptibility has not been determined. To test this hypothesis, the *hap1-Ty1* mutation was deleted in S288C strains BY4741 and FY2609 to generate BY4741 *hap1*Δ (this study) and FY2611 *hap1*Δ (40), respectively (see Table S3). The indicated strains were tested for growth in liquid cultures and through serial-dilution spot assays with and without 16 µg/mL fluconazole (Fig. 1A). Interestingly, the BY4741 strain exhibited a slight increase in fluconazole susceptibility compared with the BY4741 *hap1*Δ strain (Fig. 1A and B). We suspect that the enhanced azole susceptibility in the BY4741 strain is because of a known insertion of an in-frame *Ty1* sequence at the 3′ end of the *HAP1* open reading frame (ORF), resulting in the expression of a mutated *HAP1* that lacks 13 amino acids from its C-terminus and contains an additional 32 amino acids encoded from the *Ty1* sequence. The insertion of the *Ty1* element does not seem to affect the growth of BY4741 (*hap1-Ty1* mutation) versus FY2609 (*HAP1* WT) under untreated conditions (Fig. 1A through C; Table S1). In contrast, deletion of *HAP1* (FY2611 *hap1*Δ) showed a hypersusceptible phenotype compared with FY2609 when grown on agar plates or in liquid culture containing 16 µg/mL fluconazole (Fig. 1A and C). In addition, both the FY2611 *hap1*Δ and BY4741 *hap1*Δ strains have a similar doubling time in the presence and absence of fluconazole (Fig. 1A through C; Table S1). To our knowledge, this is the first observation that Hap1 contributes to azole susceptibility in *S. cerevisiae*. We suspect that this phenotype has not been observed until now because earlier functional genomics screens used the BY4741 and BY4742 parental and deletion strain collections (47).

### Sequence comparison, phylogenetics, and synteny of Hap1 homologs

**Fig 2:**
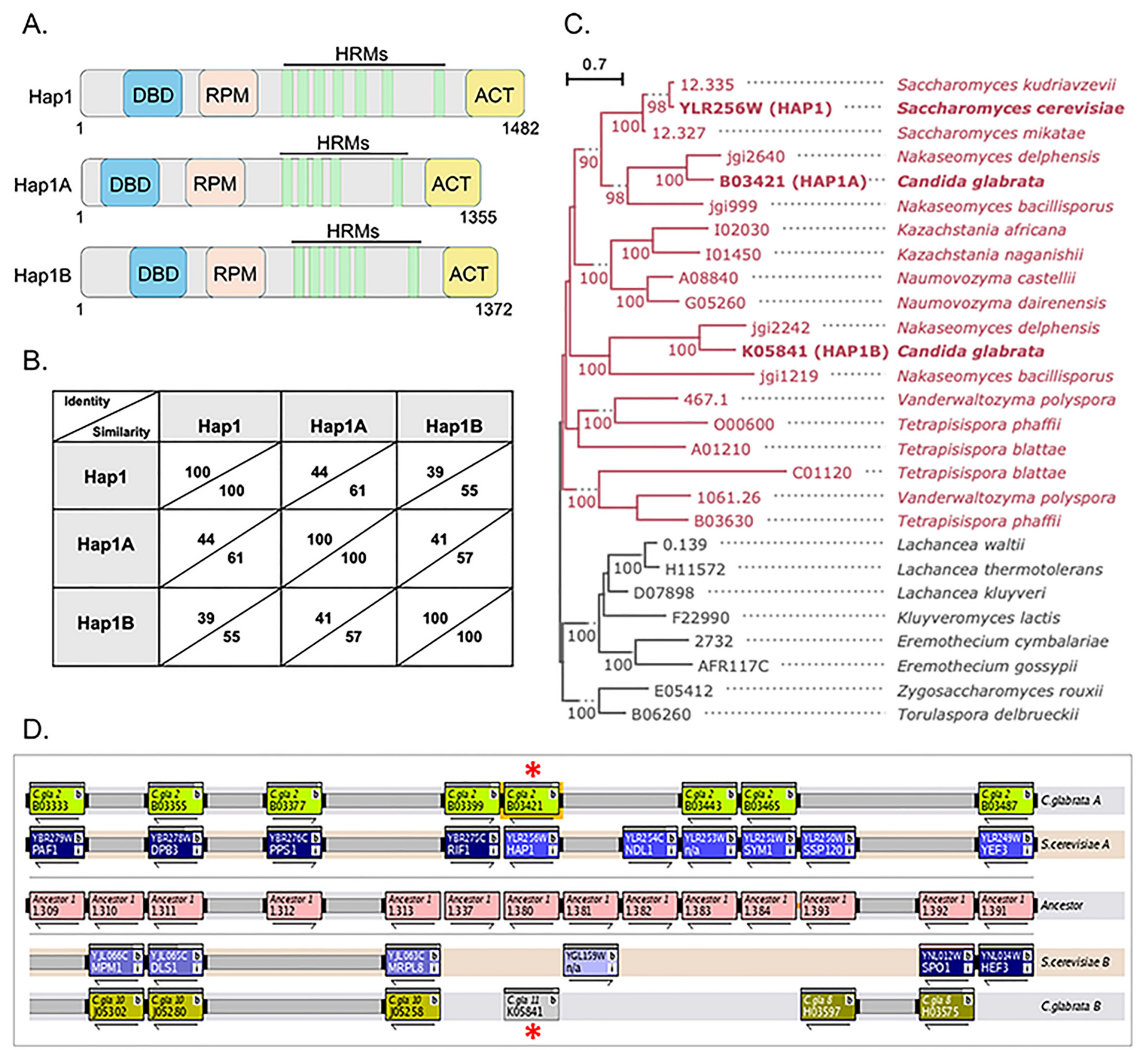
Sequence and Evolutionary analysis of Hap1. (**A**) Schematic of conserved domains found in *S. cerevisiae* Hap1, *C. glabrata*, Hap1A, and *C. glabrata* Hap1B. (**B**) Table indicating the percent identity and similarity between Hap1, Hap1A, and Hap1B. (**C**) Gene phylogeny of Hap1 homologs. The phylogeny was rooted based on the species tree, and branch values represent ultrafast bootstrap support. Red color indicates species that evolved following the whole genome duplication event in their last common ancestor, and black color indicates outgroup species. (**D**) Syntenic analysis between *S. cerevisiae HAP1* (YLR256W) and *C. glabrata* Hap1A (*CAGL0B03421*) and Hap1B (*CAGL0K05841*) provided by the Yeast Gene Order Browser (http://ygob.ucd.ie/). Red asterisks mark the position of the *HAP1*A (*CAGL0B03421g*) and HAP1B (*CAGL0K05841g*).

Two proteins that are encoded by *CAGL0B03421g* or *HAP1A* (aliases *MAR1* or *ZCF4*) and *CAGL0K05841g* or *HAP1B* (aliases *HAP1* or *ZCF27*) in *C. glabrata* share a similar domain structure with *S. cerevisiae* Hap1 (Fig. 2A). The three domain structures conserved across all three proteins include the N-terminal DNA-binding domain (DBD), a repression module (RPM), and the C-terminal activation domains (ACTs) (Fig. 1A). In addition, Hap1 contains seven heme-responsive motifs (HRMs) characterized by a (K/R)CP(V/L)DH sequence motif (48, 49), whereas Hap1A has five HRMs, and Hap1B has six HRMs (Fig. 2A). Amino acid sequence comparisons indicate that Hap1A shows a higher percent sequence identity and similarity to Hap1 than to Hap1B (Fig. 2B). Additionally, a phylogenetic tree was constructed to investigate the evolutionary relationships between Hap1, Hap1A, and Hap1B, which shows that Hap1A groups more closely with Hap1 compared to Hap1B (Fig. 2C). Furthermore, synteny information provided by the Yeast Gene Order Browser (http://ygob.ucd.ie/) (50) indicates that Hap1 and Hap1A are syntenic (Fig. 2D). Taken together, our evolutionary analysis indicates that Hap1, Hap1A, and Hap1B evolved by whole genome duplication and that Hap1 and Hap1A are orthologs. In addition, the Hap1B ortholog was lost in *Saccharomyces* by reductive evolution at some point in an ancestor of the genus, returning the gene family to a single copy status in *S. cerevisiae*.

### Hap1B, rather than, Hap1A alters azole susceptibility in *C. glabrata*

**Fig 3:**
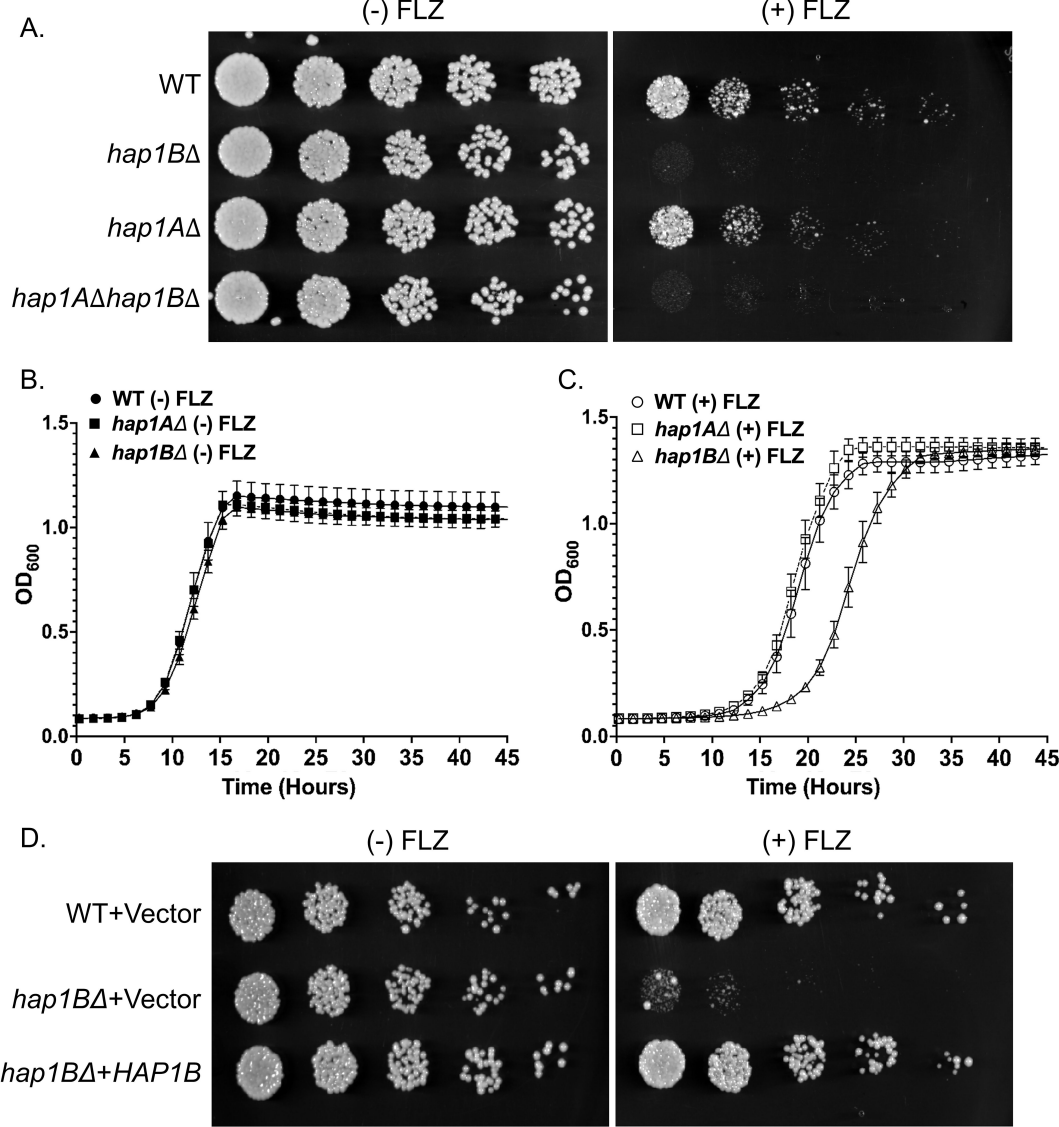
*C. glabrata* Hap1B, rather than, Hap1A alters fluconazole susceptibility. (**A**) Fivefold serial dilution spot assays of *Cg*2001 WT, *hap1B*Δ*,* and *hap1A*Δ strains plated on SC plates with and without 32 µg/mL fluconazole. A minimum of three biological replicates were performed. (**B**) Liquid growth curves of the indicated *C. glabrata* strains grown in SC media with or without 32 µg/mL fluconazole. Error bars represent SD. (**C**) Fivefold serial dilution assays of *Cg*989 WT and *hap1B*Δ transformed with plasmids expressing *HAP1B* from its endogenous promoter or empty vector spotted on SC-Ura plates with and without 32 µg/mL fluconazole at 30°C for 48 h. Error bars represent SD. A minimum of three biological replicates were performed.

Because deletion of *HAP1* in *S. cerevisiae* altered azole susceptibility, we wanted to determine if *C. glabrata* (*Cg*) strains lacking Hap1A and Hap1B have a similar susceptibility to azole drugs. To test this hypothesis, we deleted *HAP1A* and *HAP1B* in the *C. glabrata* CBS138 (ATCC *Cg*2001) WT strain and performed liquid growth and serial-dilution spot assays with and without 32 µg/mL fluconazole (Fig. 3A through C). In the untreated conditions, both *hap1B*Δ and *hap1A*Δ strains grew similar to the *Cg*2001 WT strain on agar plates and liquid cultures (Fig. 3A through C). We also did not observe any differences in doubling times (Table S2). However, in the presence of fluconazole, the *hap1B*Δ strain showed an azole hypersusceptibility phenotype on agar plates along with a growth delay and longer doubling times when cultured in liquid media, whereas *hap1A*Δ strain grew like the *Cg*2001 WT strain (Fig. 3A and C; Table S2). Furthermore, a *hap1A*Δ*hap1B*Δ double-deletion strain showed azole susceptibility similar to a *hap1B*Δ strain (Fig. 3A). To confirm that our observed azole hypersusceptible phenotype was due to the loss of *HAP1B*, complementation assays were performed using a *C. glabrata* (ATCC 200989 or *Cg*989 WT) strain. The full-length *HAP1B* open-reading frame with its endogenous promoter were cloned in the pGRB2.0 plasmid and transformed into a *Cg*989 *hap1B*Δ deletion strain (Tables S3 and S4). The pGRB2.0 vector was also transformed into the *Cg*989 WT and *hap1B*Δ strains as controls. The *HAP1B* plasmid construct was able to rescue azole susceptibility as shown by a serial-dilution spot assay (Fig. 3D), while the *hap1B*Δ strain expressing the plasmid only (Vector) construct remain hypersusceptible (Fig. 3D). In addition, gene expression analysis also confirmed that *HAP1B* and *HAP1A* were not expressed in their respective deletion strains (Fig. S1A and B). In addition, we confirmed that the genes upstream and downstream of *HAP1B* were expressed in *hap1B*Δ similar to the *Cg*2001 WT strain (Fig. S2A and B). Finally, we also deleted the upstream (*CAGL0K05819g*) and downstream (*CAGL0K05863g*) genes and observed little to no change in azole susceptibility (Fig. S2C). Overall, our data show that Hap1B, rather than Hap1A, plays a specific role in mediating azole susceptibility.

### Expression of *CYC1* depends on Hap1B, but not Hap1A, because of differences in protein expression

**Fig 4:**
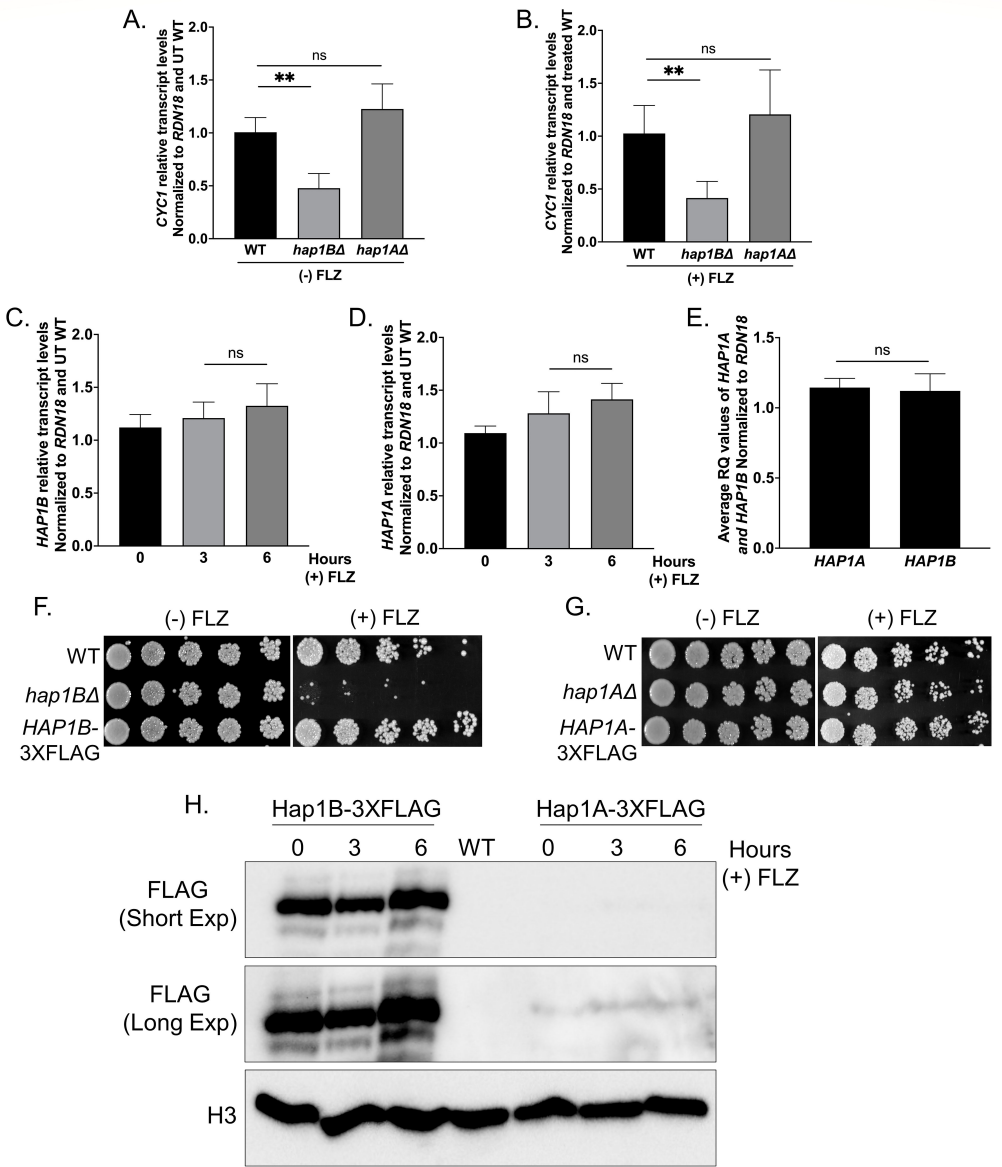
Transcript and protein analysis of *CYC1*, *HAP1B*, and *HAP1A*. (**A and B**) Transcript levels of *CYC1* from the indicated strains treated with and without 64 µg/mL fluconazole for 3 h. (**C and D**) Transcript levels of *HAP1B* and *HAP1A* from the indicated strains treated with and without 64 µg/mL fluconazole for 3 h. For panels A–D, transcript levels were set relative to WT and normalized to *RDN18* mRNA levels. (**E**) Transcript level comparison of *HAP1A* and *HAP1B* from untreated WT. Data were analyzed from three or more biological replicates with three technical replicates. Statistics were determined using the GraphPad Prism Student *t*-test, version 9.5.1. Error bars represent SD. ns, *P* > 0.05; **P < 0.01. (**F and G**) Fivefold serial dilution spot assays of *Cg*2001 WT, *hap1B*Δ, *hap1A*Δ*, HAP1B-3×FLAG, and HAP1A-3×FLAG* strains plated on SC plates with and without 32 µg/mL fluconazole. A minimum of three biological replicates were performed. (**H**) Western blot analysis of Hap1B-3× FLAG and Hap1A-3×FLAG with and without treatment with 64 µg/mL fluconazole for 3 and 6 h. Western blots showing Short (Short Exp) and long (Long Exp) chemiluminescence exposure. Histone H3 was used as the loading control.

In *S. cerevisiae*. Hap1 is known to regulate the expression of the *CYC1* gene (48, 49, 51–53). To determine if Hap1B and/or Hap1A also controls the expression of *C. glabrata CYC1* gene, *Cg*2001 WT, *hap1B*Δ, and *hap1A*Δ strains were grown in the presence and absence of azole treatment, and qRT-PCR transcript analysis was performed. Interestingly, *CYC1* transcript analysis revealed that the loss of *HAP1B*, but not *HAP1A*, resulted in a 50% decrease in *CYC1* expression, irrespective of drug treatment (Fig. 4A and B). To determine if this difference was a consequence of transcript levels of *HAP1B* and *HAP1A,* qRT-PCR analysis was performed on *Cg*2001 WT cells treated with or without 64 µg/mL fluconazole for three or 6 h. Both *HAP1B* and *HAP1A* transcript levels were expressed with no significant differences between untreated and fluconazole treated conditions (Fig. 4C through E; Table S7). Furthermore, *HAP1B* transcript levels are not altered in *hap1A*Δ strain and vice versa, indicating they are independent of each other (Fig. S1A and B). Because there were similar transcript levels between *HAP1A* and *HAP1B* (Fig. 4E), we checked for differences in protein levels between Hap1B and Hap1A. In order to do this, we constructed, and PCR confirmed 3×FLAG-tagged strains where the 3×FLAG-tag was genomically inserted at the C-terminus of *HAP1B* and *HAP1A*. We also observed no significant changes in growth between WT and *3×FLAG*-tagged strains when treated with and without 32 µg/mL azole (Fig. 4F and G). For protein extraction of the indicated strains, *HAP1B-3×FLAG* and *HAP1A-3×FLAG* tagged strains were grown with or without 64 µg/mL fluconazole for 3 or 6 h. Western blot analysis indicated that the Hap1B-3×FLAG protein expression remained nearly constant with and without drug treatment (Fig. 4H). Unexpectedly, we observed virtually no expression of Hap1A-3×FLAG protein regardless of drug treatment (Fig. 4H, Short Exp). Even with longer exposure times, barely detectable levels of Hap1A were observed (Fig. 4H, Long Exp), suggesting that Hap1A is regulated at the post-transcriptional level. Due to essentially undetectable levels of Hap1A protein, we suspect that this is why a *hap1A*Δ strain does not alter *CYC1* gene expression or show hypersusceptibility to azoles.

### Hap1B is dispensable for expression of drug efflux pumps but is needed for azole-induced expression of ergosterol (*ERG*) genes

**Fig 5:**
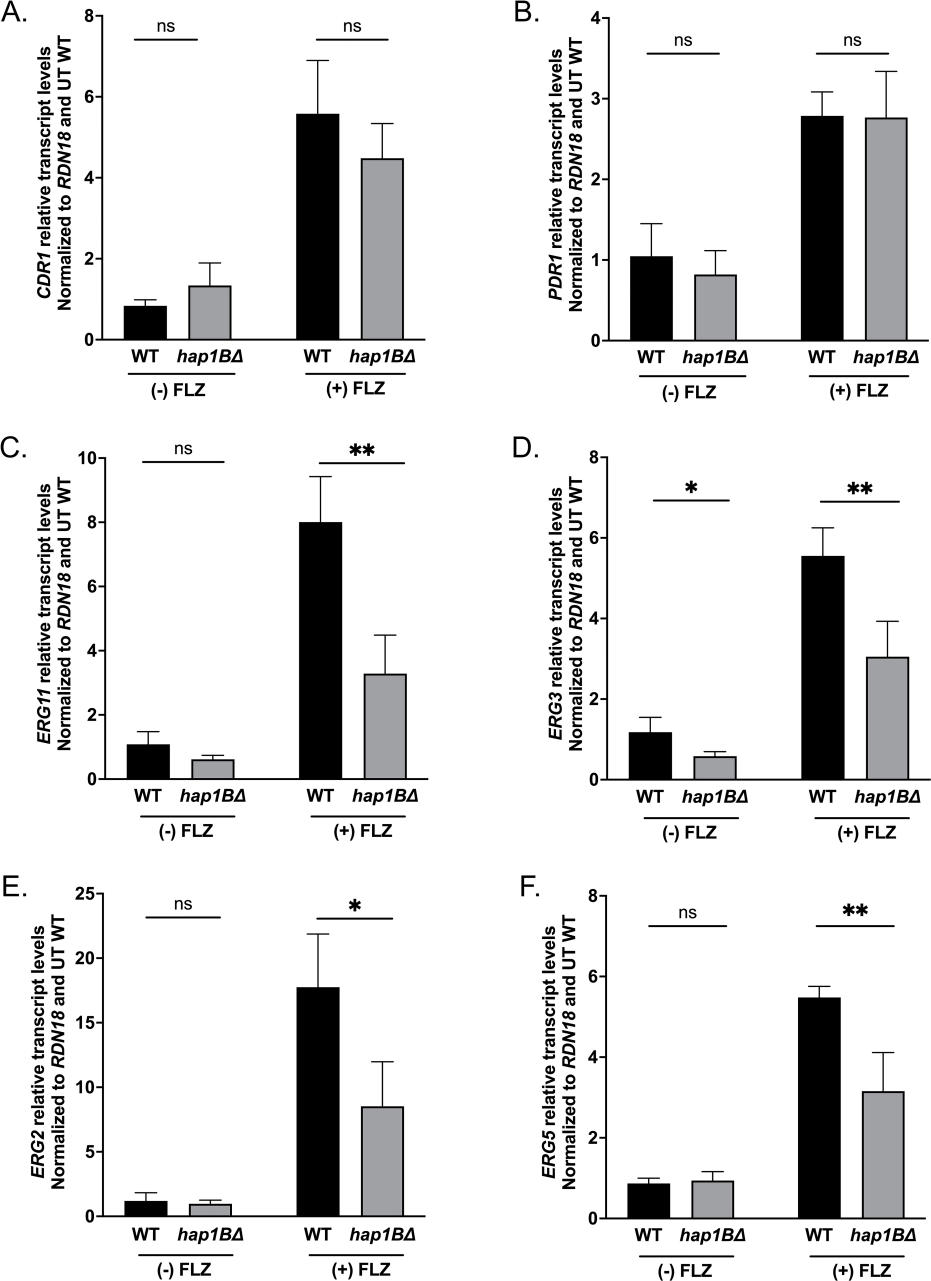
Transcript analysis of drug efflux pump and ergosterol (*ERG*) genes. (**A and B**) Transcript levels of drug efflux pumps in *Cg*2001 WT and *hap1B*Δ strains treated with or without 64 µg/mL fluconazole for 3 h. (**C–F**) Transcript levels of *ERG* genes in *Cg*2001 WT and *hap1B*Δ strains treated with or without 64 µg/mL fluconazole for 3 h. For panels A–F, all strains were treated with or without 64 µg/mL fluconazole for 3 h. Transcript levels were set relative to the untreated WT and normalized to *RDN18* mRNA levels. Data were analyzed from four biological replicates with three technical replicates each. Statistics were determined using the GraphPad Prism Student *t* test, version 9.5.1. ns, *P* > 0.05; *, *P* < 0.05; **, *P* < 0.01. Error bars represent the SD.

Because the *hap1B*Δ strain showed altered azole susceptibility (Fig. 3A through C), we wanted to identify the mechanism mediating this phenotype. A common mechanism of altering azole resistance in *C. glabrata* involves the upregulation of drug efflux pumps, such as *CDR1*, *PDH1*, and *SNQ2*, facilitated by the zinc cluster transcription factor Pdr1 (28, 29, 31, 54, 55). To determine if expression of drug efflux pumps is altered in the *hap1B*Δ strain in the presence or absence of 64 µg/mL fluconazole, the expression levels of the known azole transporters *CDR1, PDH1,* and *SNQ2* as well as the transcriptional regulator *PDR1* were analyzed by qRT-PCR analysis. Our transcript analysis revealed no significant difference in the expression of any of the genes encoding ABC transporters in the *hap1B*Δ strain compared with the *Cg*2001 WT strain (Fig. 5A and B; Fig. S3A and B), indicating that altered expression of known azole drug efflux pumps under these conditions is not the main reason for azole hypersusceptibility for the *hap1B*Δ strain.

In *S. cerevisiae*, Hap1 is known to regulate steady state transcript levels of ergosterol biosynthesis genes, such as *ERG11*, *ERG3*, *ERG5*, and *ERG2* (40, 41, 44, 49, 52, 56, 57). In addition, altered *ERG11* gene expression in *C. glabrata* is also a mechanism that can lead to azole hypersusceptibility phenotypes (24, 58, 59). To determine if altered *ERG* gene expression was a mechanism for the observed azole hypersusceptibility of the *hap1B*Δ strain, *Cg*2001 WT and *hap1B*Δ strains were treated with and without 64 µg/mL fluconazole, and *ERG11*, *ERG3*, *ERG5,* and *ERG2* transcript levels were analyzed by qRT-PCR. In the absence of the drug, with the exception of *ERG3*, no significant difference in the expression levels of *ERG11*, *ERG5,* or *ERG2* was observed between the *Cg*2001 WT and *hap1B*Δ strains (Fig. 5C through F). However, upon treatment with fluconazole, all four *ERG* genes failed to induce to wild-type levels in the *hap1B*Δ strain (Fig. 5C through F). Furthermore, a *hap1A*Δ strain did not have altered *ERG11* and *ERG3* expression, which coincides with its lack of expression and azole hypersusceptible phenotype (Fig. S3C and D). Altogether, our data indicate that in addition to Upc2A, Hap1B serves as another critical transcription factor for the azole-induced expression of the late ergosterol pathway genes.

### Hap1B-3×FLAG is enriched at *ERG* gene promoters

**Fig 6:**
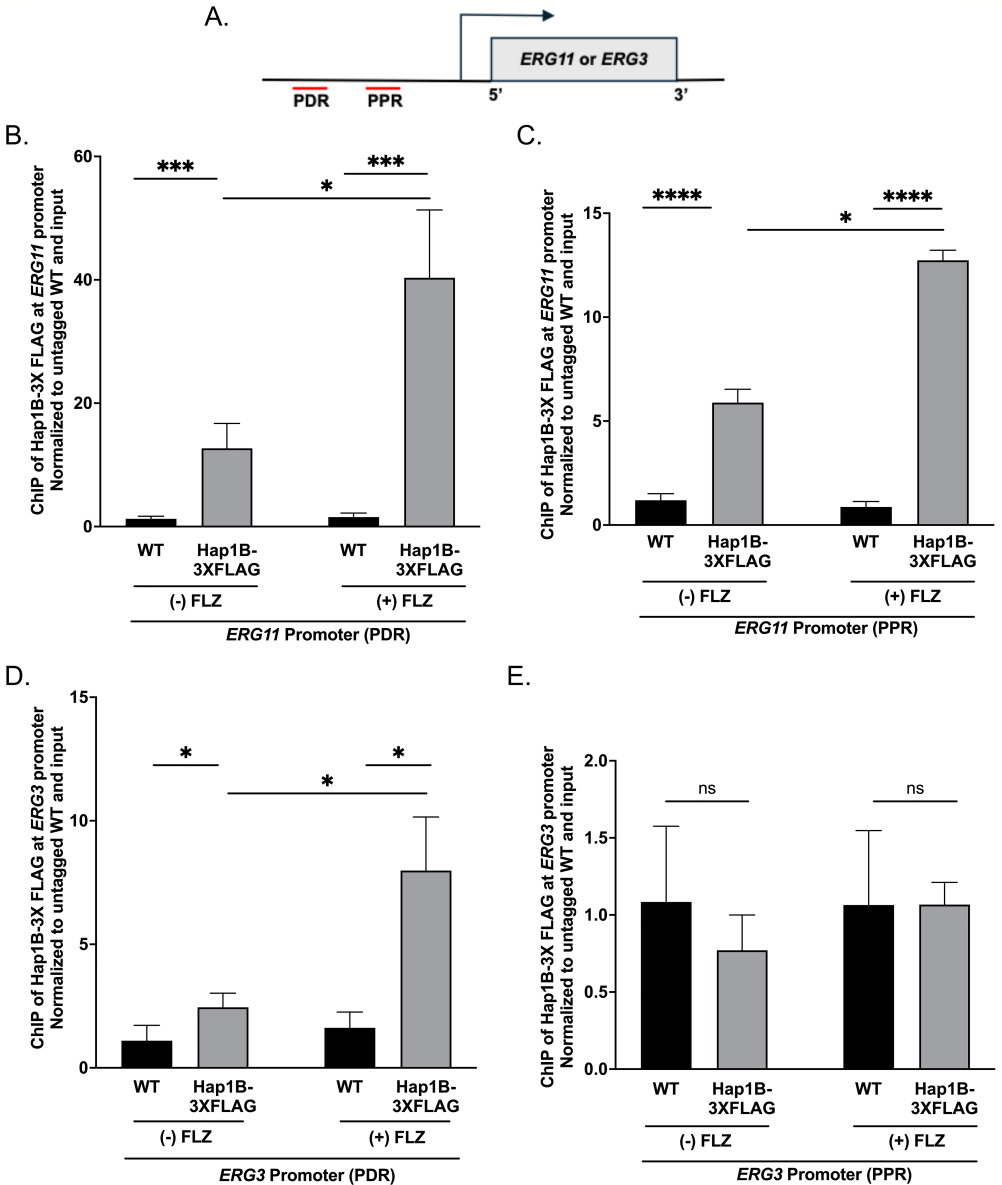
Chromatin immunoprecipitation analysis of Hap1B at *ERG* gene promoters. (**A**) Schematic of *ERG11* and *ERG3* promoter distal region (PDR) and promoter proximal region (PPR). (**B–E**) ChIP analysis of *Cg*2001 WT (untagged), Hap1B-3×FLAG at two *ERG11* promoter regions (PDR and PPR), and two *ERG3* promoter regions (PDR and PPR) when treated with or without 64 µg/mL fluconazole for 3 h. ChIP analysis was normalized to DNA input samples and set relative to untagged WT. Statistics were determined using the GraphPad Prism Student *t* test, version 9.5.1. ns, *P* > 0.05; *, *P* < 0.05; ***, *P* < 0.001; ****, *P* < 0.0001. Error bars represent SD for three biological replicates with three technical replicates.

Because our data show decreased expression of ergosterol genes in the *hap1B*Δ strain upon azole treatment (Fig. 5C through F), we suspect that Hap1B is a direct transcription factor for the *ERG* genes. To determine if Hap1B directly targets the promoter of the *ERG11* gene, chromatin immunoprecipitation (ChIP) assays were performed using anti-FLAG monoclonal antibodies and chromatin isolated from untagged *Cg*2001 WT and Hap1B-3×FLAG strains, treated with or without fluconazole. ChIP-qPCR fluorescent probes were designed to recognize a promoter distal region (PDR) and promoter proximal region (PPR) of the *ERG11* promoter (Fig. 6A). Using these probes, a significant enrichment of Hap1B was detected at both *ERG11* promoter regions compared to the untagged control (Fig. 6B and C; Table S8). In addition, Hap1B was further enriched at the promoter of *ERG11* upon azole treatment (Fig. 6B and C; Table S8) supporting the importance of Hap1B in azole-induced gene expression. No significant enrichment of Hap1B was detected at the 3′ UTR of *ERG11* regardless of treatment (Fig. S4), indicating specific enrichment at the promoter region.

We also examined Hap1B localization status on the *ERG3* promoter by ChIP analysis (Fig. 6D and E). To determine if Hap1B binds to the promoter of *ERG3,* two ChIP-qPCR fluorescent probes were designed to recognize the promoter distal region (PDR) and promoter proximal region (PPR) of the *ERG3* promoter (Fig. 6A). Similar to the *ERG11* promoter, Hap1B was detected at the *ERG3* distal promoter region and was further enriched upon fluconazole treatment (Fig. 6D; Table S8). However, we did not detect any Hap1B enrichment at the proximal promoter region (Fig. 6E; Table S8) regardless of azole treatment. Overall, our data demonstrate that Hap1B directly targets the promoters of *ERG11* and *ERG3* to help facilitate the proper expression of *ERG* genes and maintenance of ergosterol homeostasis during azole treatment.

### The *hap1B*Δ strain has altered azole susceptibility due to decreased ergosterol levels, which can be suppressed by exogenous sterols and active sterol import

**Fig 7:**
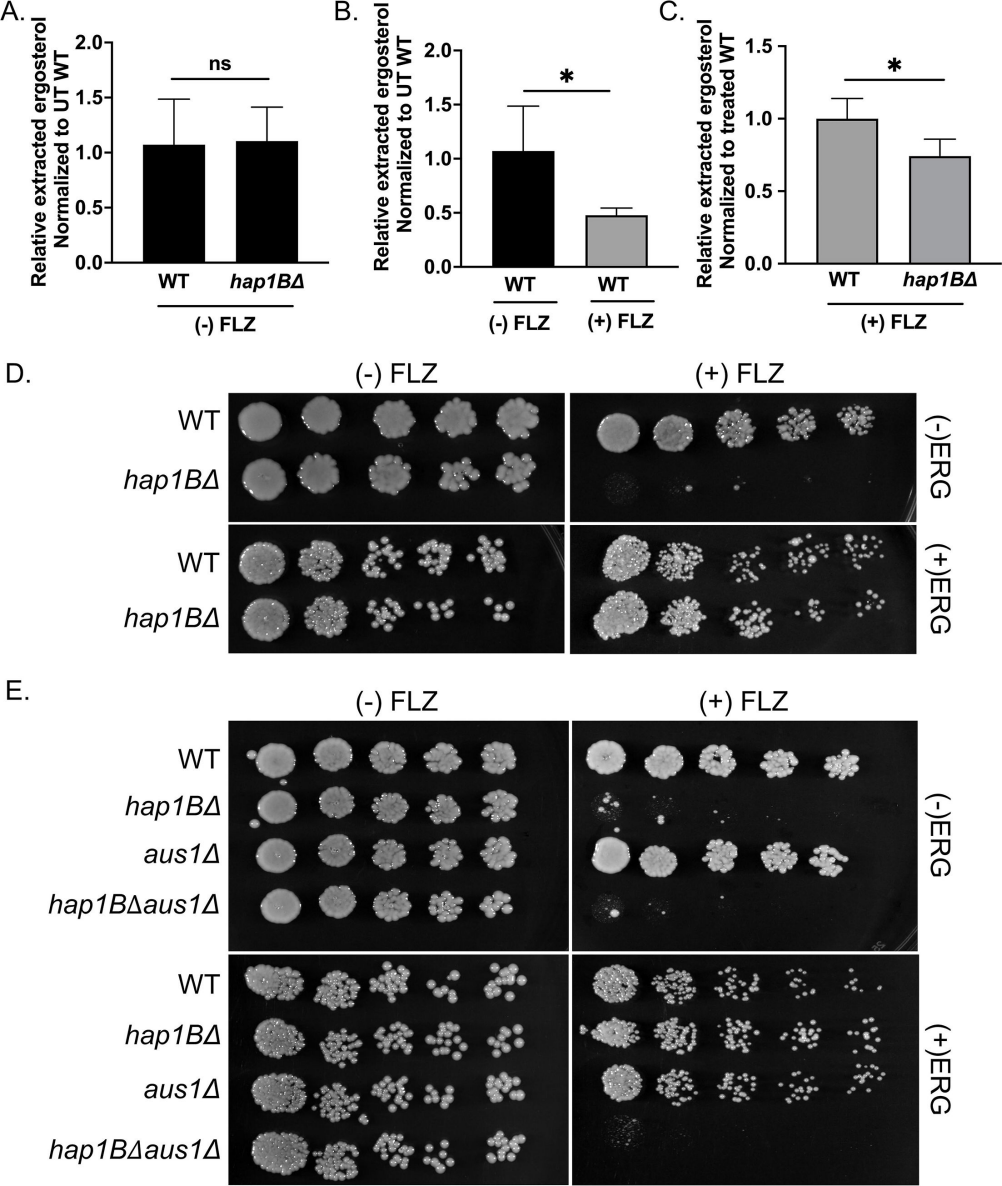
Disruption of ergosterol levels in a *hap1B*Δ strain alters azole susceptibility (**A–C**) HPLC analysis of the total ergosterol extracted from the *Cg*2001 WT and *hap1B*Δ strains treated with or without 64 µg/mL fluconazole for 3 h. The figure represents a ratio between ergosterol and cholesterol compared with treated and untreated WT samples. Data were generated from four biological replicates. Statistics were determined using the GraphPad Prism Student *t* test, version 9.5.1. ns, *P* > 0.05; *, *P* < 0.05. Error bars represent the SD. (**D and E**) Fivefold dilution spot assays of the indicated *C. glabrata* strains grown on SC plates with and without 32 µg/mL fluconazole and/or with and without 20 µg/mL ergosterol. A minimum of three biological replicates were performed.

Because azole-induced *ERG* gene expression is diminished in the *hap1B*Δ strain, we would expect an additional decrease in ergosterol levels in this strain, which would explain why a *hap1B*Δ strain has an increase in azole susceptibility. To ascertain whether total endogenous ergosterol levels differed between *Cg*2001 WT and *hap1B*Δ strains upon azole treatment, non-polar lipids were extracted from both strains in the presence and absence of 64 µg/mL fluconazole. Total ergosterol level was measured by high-performance liquid chromatography (HPLC) analysis, and cholesterol was used as an internal standard control. No significant difference was observed between *Cg*2001 WT and *hap1B*Δ strains in the untreated conditions (Fig. 7A), concurring with our gene expression analysis showing no significant difference in expression of multiple *ERG* genes without azole treatment. However, upon fluconazole treatment, the *Cg*2001 WT strain demonstrated the expected decrease in ergosterol levels (Fig. 7B), whereas the *hap1B*Δ strain exhibited an additional 30% reduction in total ergosterol compared with the treated *Cg*2001 WT strain (Fig. 7C).

Due to this observation, we hypothesized that the decrease in ergosterol content contributes to azole hypersensitivity and reasoned that exogenous supplementation with ergosterol would suppress the azole hypersensitive phenotype observed for the *hap1B*Δ strain. To test this hypothesis, *Cg*2001 WT and *hap1B*Δ strains were plated on synthetic complete (SC) media supplemented with or without exogenous ergosterol and/or fluconazole. In support of our hypothesis, serial-dilution spot assays showed that the addition of exogenous ergosterol completely suppressed the azole hypersensitive phenotype of the *hap1B*Δ strain, whereas *hap1B*Δ strain without ergosterol retained the hypersensitive phenotype (Fig. 7D). Because ergosterol is solubilized in the presence of Tween 80–ethanol solution, we wanted to determine if this suppression was specific to ergosterol. Thus, *Cg*2001 WT and *hap1B*Δ strains were plated on SC media supplemented with a Tween 80–ethanol solution with or without fluconazole. As indicated in Fig. S5, Tween 80–ethanol did not suppress *hap1B*Δ azole hypersusceptible phenotype (Fig. S5) indicating that suppression was mediated by exogenous ergosterol uptake.

Based on these observations, we also expected that deletion of the only known sterol importer *AUS1* would prevent sterol uptake by *hap1B*Δ strains (60–62). To determine this, we constructed an *aus1*Δ strain and a *hap1B*Δ*aus1*Δ double deletion strain and performed serial-dilution spot assays on agar plates supplemented with or without exogenous ergosterol in the presence and/or absence of fluconazole (Fig. 7E). As anticipated, the *hap1B*Δ*aus1*Δ strain remained hypersensitive to fluconazole with or without exogenous ergosterol (Fig. 7E). However, growth of the *hap1B*Δ strain was not altered by fluconazole and/or exogenous ergosterol and grew similar to the *Cg*2001 WT strain (Fig. 7E). Overall, our data elucidate the mechanistic basis and pathway underlying the hypersensitive phenotype observed in the *hap1B*Δ strain. Because Hap1A is not expressed, it is unclear what role it plays, if any, under azole treatment. In summary, our findings represent the first characterization of Hap1B as direct transcription factor for regulating ergosterol genes and ergosterol homeostasis in response to azole drug treatment.

### Hap1A is induced upon hypoxic exposure

In aerobic conditions, *S. cerevisiae* Hap1 functions as a transcriptional activator of *CYC1* and *ERG* genes (40, 41, 44, 48, 49, 51–53, 56, 57). Furthermore, our presented data suggest that Hap1B operates similarly to Hap1, by regulating the corresponding conserved genes in *C. glabrata*. Interestingly, in *S. cerevisiae*, Hap1 functions also as a transcriptional repressor to shut down *ERG* genes under hypoxia by recruiting a corepressor complex containing Set4, Tup1, and Ssn6 corepressors (40, 57, 63, 64). Currently, it is not known if Hap1B, Hap1A, or another transcription factor functions to repress *C. glabrata ERG* genes under hypoxic conditions.

**Fig 8:**
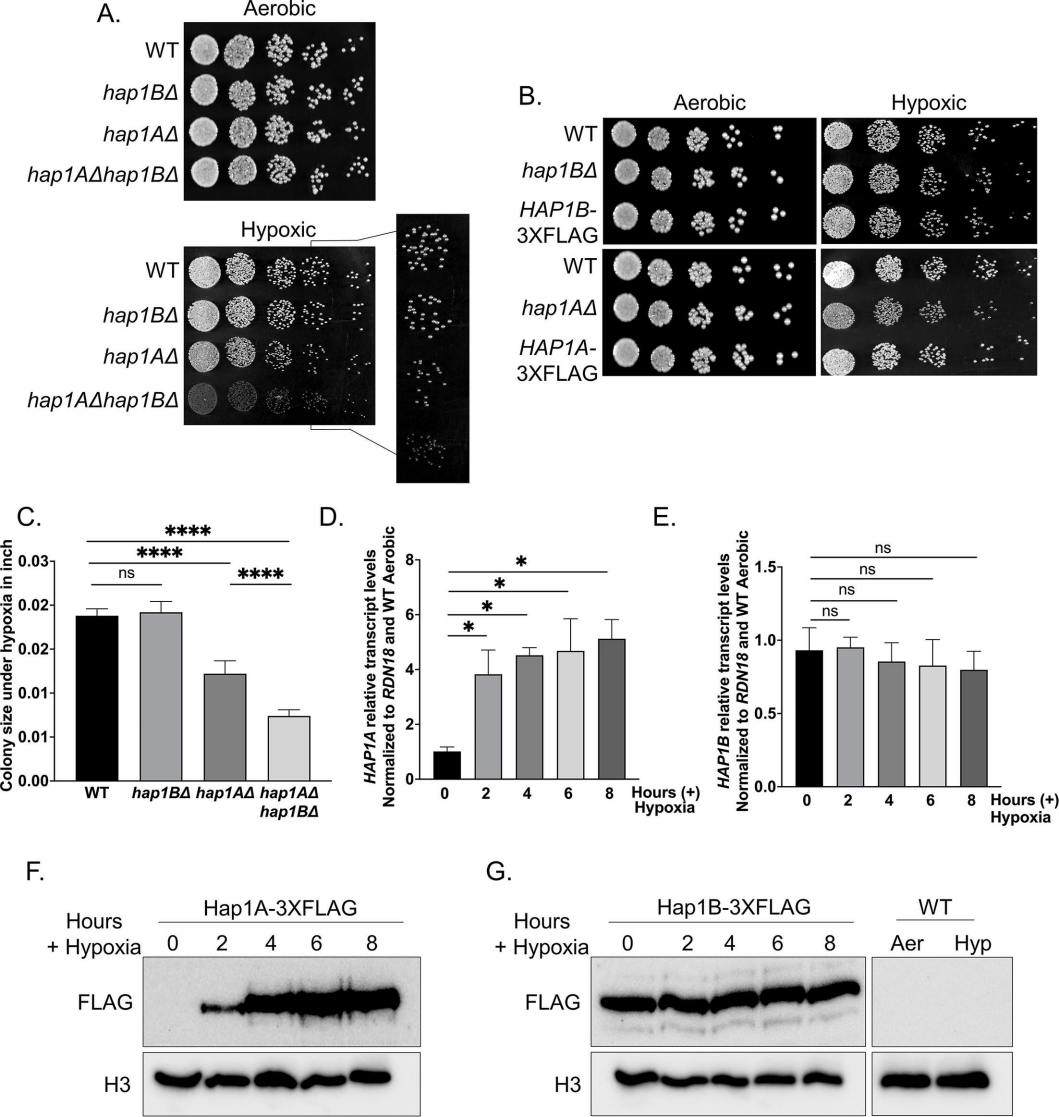
Phenotypic and expression analysis of *C. glabrata* strains under hypoxic conditions (**A and B**) Fivefold serial dilution spot assays of *Cg*2001 WT, *hap1B*Δ, *hap1A*Δ*, HAP1B-3×FLAG, and HAP1A-3×FLAG* strains grown on YPD plates under aerobic and hypoxic conditions. The fourth dilution of the hypoxic plate (A) was enlarged for enhanced visibility. A minimum of three biological replicates were performed. (**C**) Graphical representation of colony sizes of the indicated strains when grown under hypoxic conditions. Colony sizes were measured using ImageJ, version 1.51. Statistics were determined using the GraphPad Prism Student *t* test, version 9.5.1. ns, *P* > 0.05; ***, *P* < 0.001. (**D and E**) Transcript analysis of *HAP1A* and *HAP1B* of the *Cg*2001 WT strain when grown under hypoxic conditions over a time course of 0, 2, 4, 6, and 8 h. The relative transcript levels were set to *Cg*2001 WT before hypoxic exposure (0 hr) and normalized to *RDN18*. Statistics were determined using the GraphPad Prism Student *t* test, version 9.5.1. ns, *P* > 0.05; *, *P* < 0.05. (**F and G**) Western blot analysis of Hap1A-3×FLAG and Hap1B-3×FLAG protein levels over a time course of 0, 2, 4, 6, and 8 h of hypoxic exposure. *Cg*2001 WT (untagged) strain was used as a negative control for both aerobic (Aer) and hypoxic (Hyp) conditions. Histone H3 was used as a loading control.

Due to our observed phenotype for the *hap1B*Δ strain, but not for the *hap1A*Δ strain under azole treated conditions, *C. glabrata Cg*2001 WT, *hap1B*Δ, and *hap1A*Δ strains were serially diluted fivefold on agar plates and grown under aerobic or hypoxic conditions (Fig. 8A). Interestingly, under hypoxic conditions, *hap1A*Δ and *hap1A*Δ*hap1B*Δ but not *hap1B*Δ strains exhibited a statistically significant slow growth defect, as determined by colony size (Fig. 8A). We also did not observe any significant changes in growth difference between WT and *3×FLAG*-tagged strains when grown under hypoxic conditions (Fig. 8B). Measuring the colony diameter revealed an approximate 40% decrease in the size of *hap1A*Δ when compared to the *Cg*2001 WT and the *hap1B*Δ colonies, suggesting a potential function for Hap1A (Fig. 8C). Interestingly, *hap1A*Δ*hap1B*Δ colonies showed a ~60% decrease in size relative to the *Cg*2001 WT and a ~38% decrease in size when compared with the *hap1A*Δ colonies (Fig. 8C), indicating that Hap1B has a compensatory function when Hap1A is absent. Due to the significant differences in protein expression observed between Hap1B and Hap1A under aerobic conditions, we also evaluated the transcript and protein expression levels of Hap1A and Hap1B under hypoxic conditions. Using qRT-PCR analysis, a fourfold increase in *HAP1A* transcript levels was detected after two hours under hypoxic conditions while *HAP1B* transcript levels remained unaltered from aerobic to hypoxic conditions (Fig. 8D and E). In addition, we assessed the protein levels of Hap1A-3×FLAG and Hap1B-3×FLAG-tagged strains using Western blot analysis. Remarkably, we detected robust levels of Hap1A protein under hypoxic conditions, while Hap1B protein levels remained the same from aerobic to hypoxic conditions (Fig. 8F and G). Taken together, we have identified Hap1A as the first hypoxia-inducible transcription factor in *C. glabrata*. Given that *S. cerevisiae* Hap1 is required for repressing *ERG* genes under hypoxic conditions, we anticipate that Hap1A is hypoxia-induced to function in a similar manner.

### Ergosterol genes are downregulated upon hypoxic conditions

**Fig 9:**
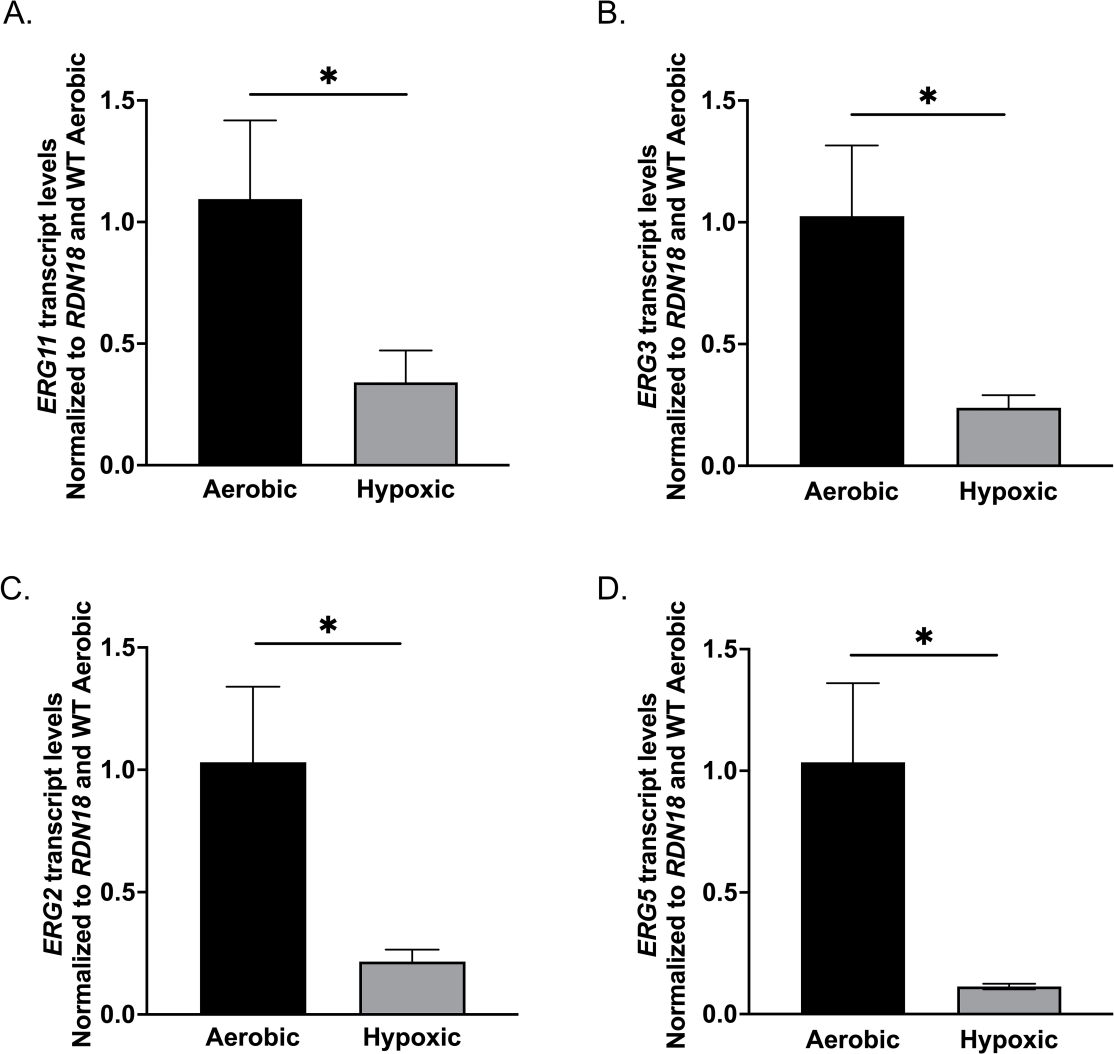
Ergosterol biosynthesis genes in *C. glabrata* are repressed upon hypoxic exposure. (**A–D**) The expression of *ERG11*, *ERG3*, *ERG2*, and *ERG5* was analyzed in *C. glabrata* WT cells under both aerobic and hypoxic conditions. Transcript analysis was set relative to aerobic WT and normalized to *RDN18* as the internal control. Data were collected from a minimum of three biological replicates, each with three technical replicates. Statistics were determined using the GraphPad Prism Student *t* test, version 9.5.1. ns, *P* > 0.05; *, *P* < 0.05. Error bars represent the SD.

In *S. cerevisiae,* it is well established that exposure to hypoxia leads to the repression of the *ERG* pathway (40, 57, 63). To determine if hypoxia-mediated repression of *ERG* genes is conserved and robust in *C. glabrata*, as observed in *S. cerevisiae*, we performed transcript analysis of multiple *ERG* genes involved in the late ergosterol biosynthesis pathway, namely, *ERG11*, *ERG3, ERG2*, and *ERG5*. When comparing the indicated *ERG* gene transcript levels under aerobic versus hypoxic conditions, we observed a significant decrease of 70%–90% in expression under hypoxic conditions (Fig. 9A through D). These findings confirm that a conserved mechanism between *S. cerevisiae* and *C. glabrata* is maintained for shutting down ergosterol biosynthesis in response to hypoxic conditions.

### Hap1A represses genes from ergosterol pathway under hypoxic conditions while Hap1B plays a compensatory role

**Fig 10:**
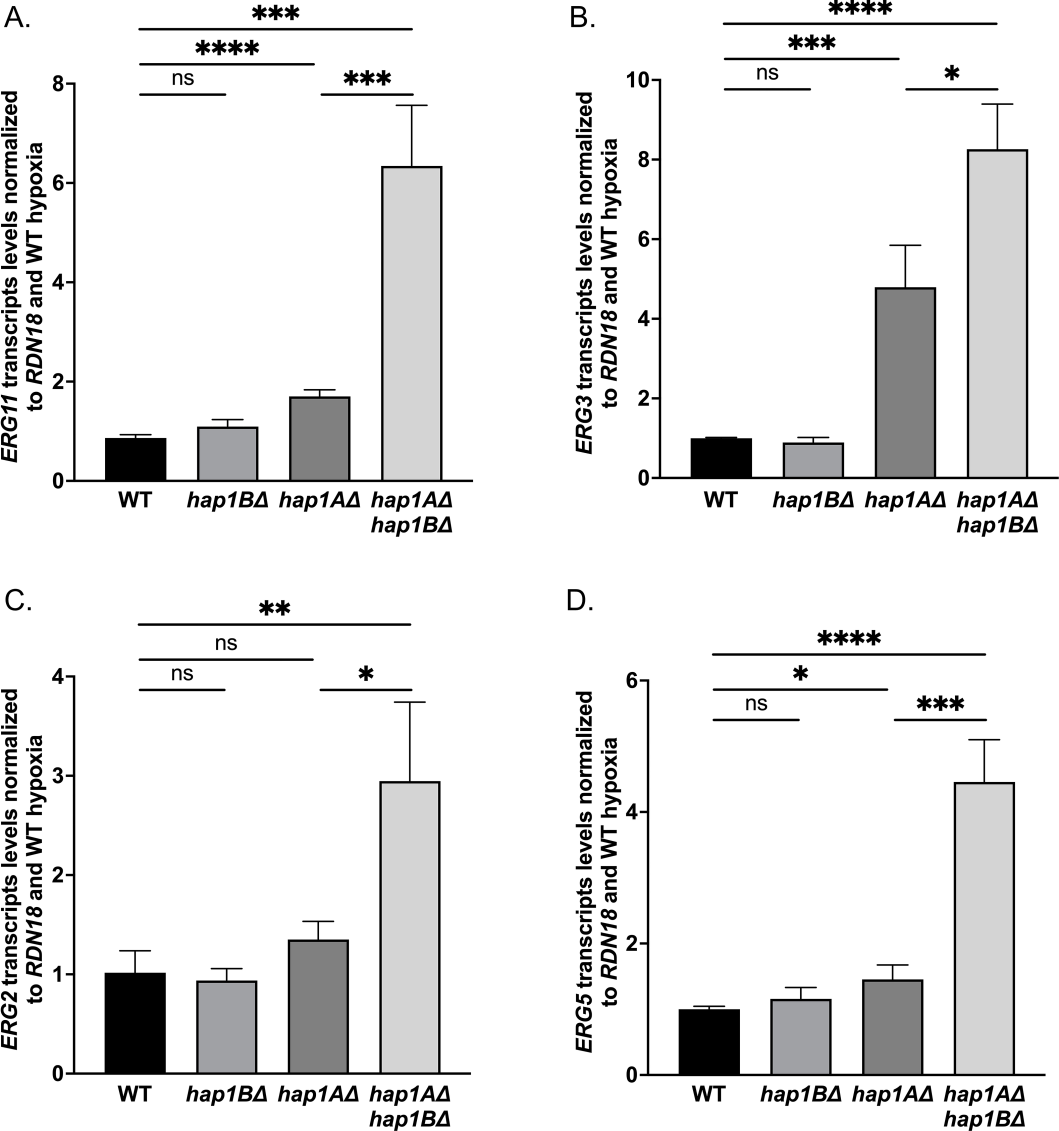
*ERG* genes are repressed by Hap1A rather than Hap1B upon hypoxic conditions. (**A–D**) After 8 h of hypoxic exposure, transcript levels of *ERG11*, *ERG3*, *ERG5,* and *ERG2* from the indicated strains were determined by qRT-PCR analysis. The levels of *ERG* genes were set relative to WT and normalized to *RDN18*. Data were generated from a minimum of three biological replicates. Statistics were determined using the GraphPad prism student *t* test, version 9.5.1. ns, *P* > 0.05; *, *P* < 0.05; **, *P* < 0.01; ***, *P* < 0.001. Error bars represent the SD.

In *S. cerevisiae,* it is known that following exposure to hypoxia *ERG* genes are repressed by a WT copy of *HAP1* but not by *hap1-Ty1* expressed in S288C strains (40, 57, 63). To determine if Hap1B and/or Hap1A shares the same function as Hap1 under hypoxic conditions, qRT-PCR analysis on *ERG* genes was performed. Surprisingly, our transcript analysis did not detect any significant differences in the transcript levels of *ERG11*, *ERG3*, *ERG5,* and *ERG2* between the *Cg*2001 WT and *hap1B*Δ strain under hypoxic conditions (Fig. 10A through D). In contrast, we observed a significant increase in the transcript levels of *ERG11*, *ERG3*, and *ERG5* genes in the *hap1A*Δ strain compared with *Cg*2001 WT strain (Fig. 10A, B and D), indicating Hap1A acts as a transcriptional repressor. *ERG2* showed no significant difference in the transcript levels upon hypoxic exposure in either *hap1B*Δ or *hap1A*Δ strain (Fig. 10C), despite being repressed upon hypoxic exposure (Fig. 9C). Interestingly, all four *ERG* genes show significant increase in transcript levels in the *hap1A*Δ*hap1B*Δ double-deletion strain when compared with the *Cg*2001 WT and *hap1A*Δ strains, suggesting that Hap1B plays a compensatory role to repress *ERG* genes (Fig. 10A through D). Overall, our findings suggest that Hap1A is directly or indirectly involved in hypoxia-induced *ERG* gene repression and suggest that Hap1B can play a compensatory role.

### Both Hap1A-3×FLAG and Hap1B-3×FLAG are enriched on *ERG11* and *ERG3* gene promoter upon hypoxic exposure

**Fig 11:**
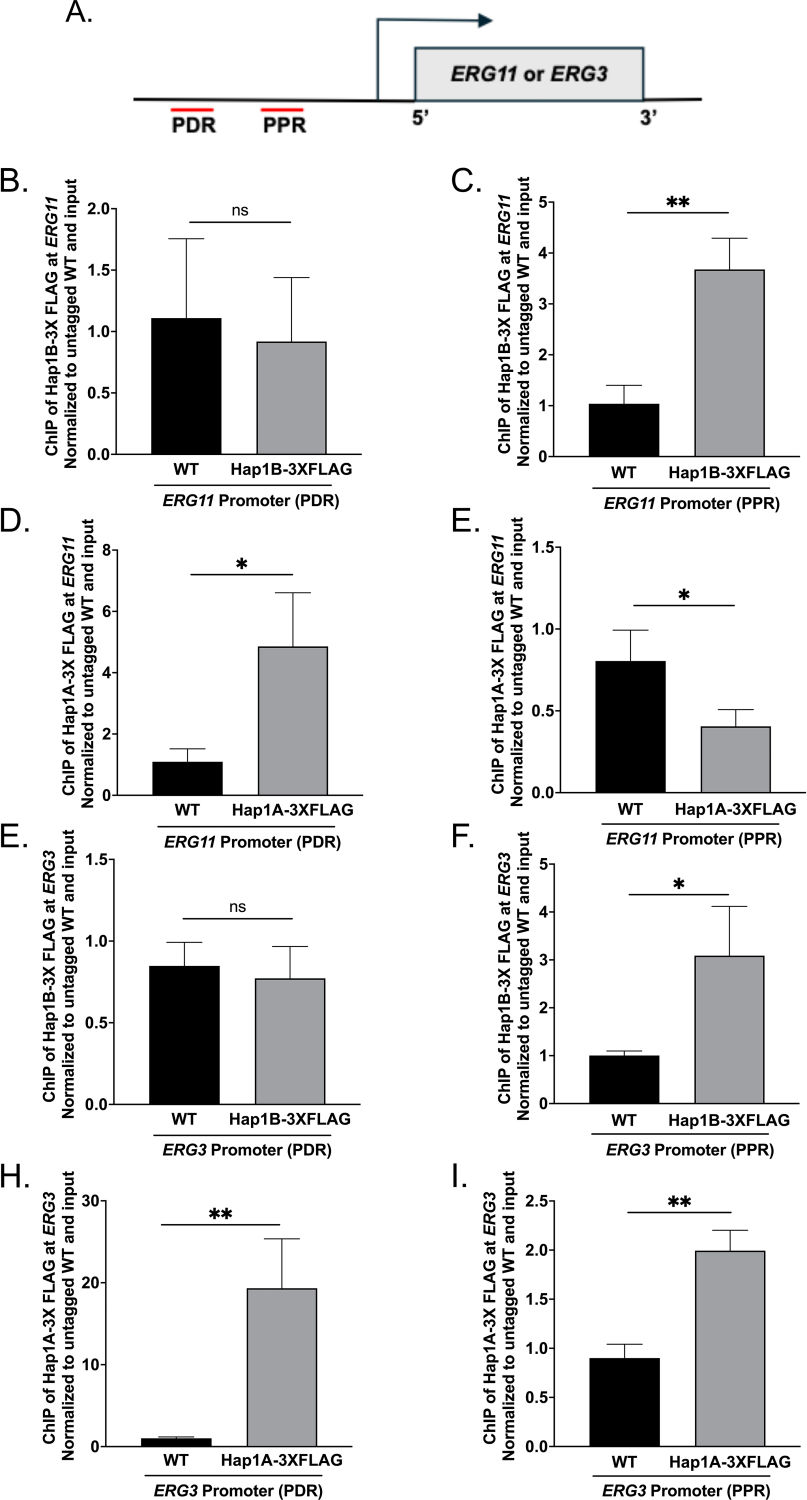
Chromatin immunoprecipitation analysis of Hap1B and Hap1A at *ERG* gene promoters under hypoxic conditions. (**A**) Schematic of *ERG11* and *ERG3* promoter distal region (PDR) and promoter proximal region (PPR). (**B–I**) ChIP analysis of *Cg*2001 WT (untagged), Hap1B-3×FLAG and Hap1A-3×FLAG at two *ERG11* promoter regions (PDR and PPR) and two *ERG3* promoter regions (PDR and PPR) after 8 h of hypoxic treatment. ChIP analysis was normalized to DNA input samples and set relative to untagged WT. Data were generated from three biological replicates, with three technical replicates each. Statistics were determined using the GraphPad Prism Student *t* test, version 9.5.1. ns, *P* > 0.05; *, *P* < 0.05; **, *P* < 0.01. Error bars represent the SD.

Because we determined that Hap1B was enriched at the promoter sequences of *ERG11* and *ERG3* under aerobic azole conditions, we wanted to assess the direct binding of Hap1B and Hap1A at *ERG* gene promoters under hypoxic conditions. To determine this, ChIP assays were performed using anti-FLAG monoclonal antibodies and chromatin isolated from untagged *Cg*2001 WT, Hap1B-3×FLAG, and Hap1A-3×FLAG strains grown for 8 h under hypoxic conditions. The same ChIP-qPCR fluorescent probes used under azole-treated conditions were utilized to assess the enrichment of Hap1B and Hap1A at the *ERG11* and *ERG3* promoter distal region (PDR) and promoter proximal region (PPR) (Fig. 11A). At the *ERG11* promoter, Hap1B was not enriched at the more distal promoter region but showed 3.5-fold enrichment at the proximal promoter region (Fig. 11B and C). Interestingly, this differs from our observations under azole-treated conditions, where Hap1B was more enriched at the distal promoter region than the proximal promoter region (Fig. 6B and C). For Hap1A, we observed a fivefold enrichment at the *ERG11* distal promoter region compared with untagged *Cg*2001 WT strain, but no enrichment was observed at the proximal region region (Fig. 11D and E). In addition, Hap1B and Hap1A enrichment was specific to the promoter of *ERG11* since no significant enrichment was observed at the 3′ UTR of *ERG11* (Fig. S6A and B). At the *ERG3* promoter, Hap1B was not enriched at the distal promoter region but showed a threefold enrichment at the proximal promoter region (Fig. 11E and F). Again, this differs from our observations under azole-treated conditions where Hap1B enriches exclusively at the *ERG3* distal promoter region but not at the proximal promoter region (Fig. 6D and E). In contrast, under hypoxic conditions, Hap1A was 20-fold enriched at the *ERG3* distal promoter region, but twofold enriched at the proximal promoter region, suggesting that Hap1A occupies both sites but prefers the distal proximal regions (Fig. 11G and H). Altogether, our data suggest that both Hap1A and Hap1B can bind to the *ERG* gene promoters under hypoxic conditions; however, each *ERG* gene may have a similar but also distinct mechanism for gene repression.

## DISCUSSION

**Fig 12:**
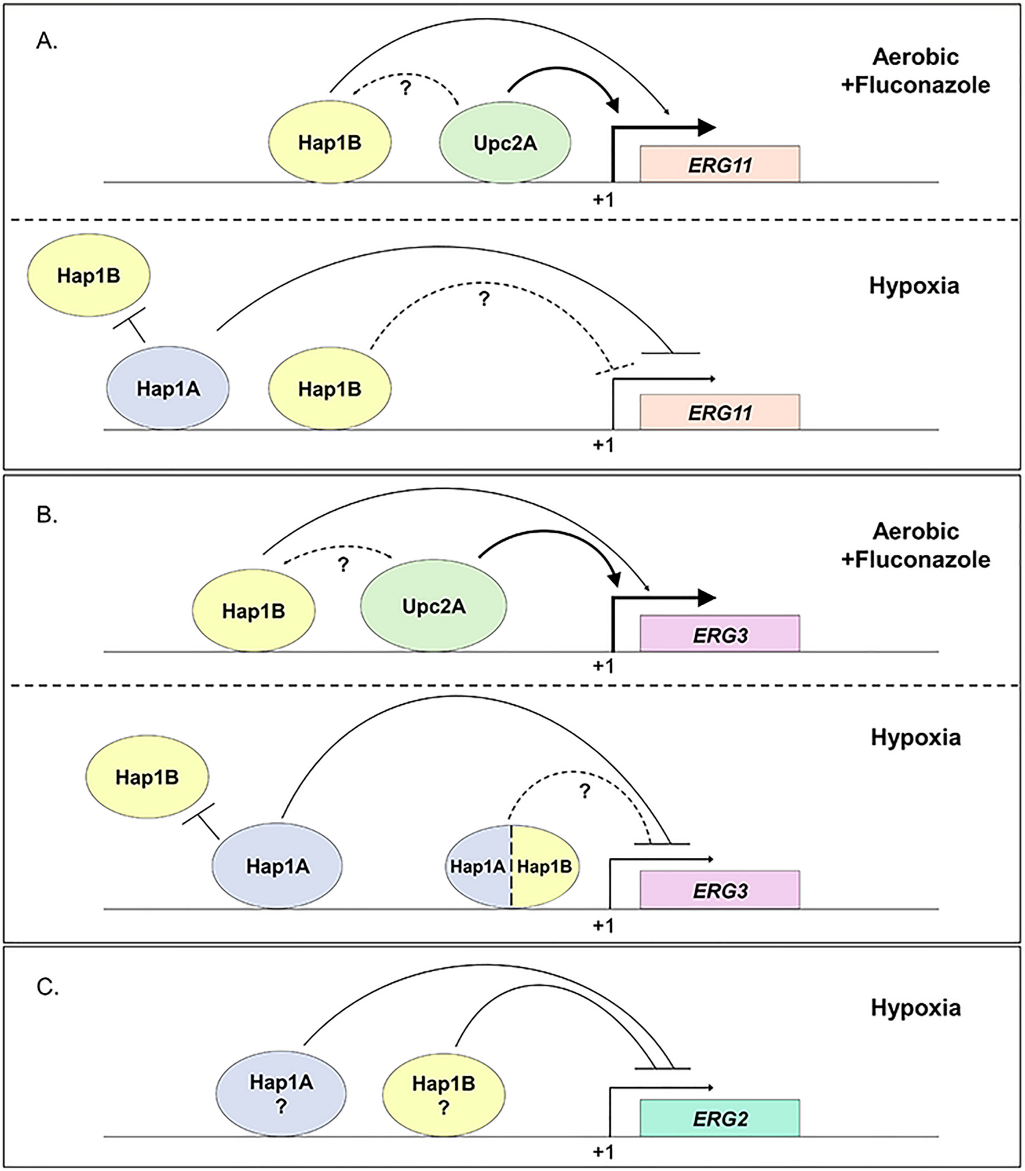
Models depicting the role of Hap1B and Hap1A in response to azole drug treatment and hypoxic conditions. (**A**) Under aerobic conditions with fluconazole treatment, Hap1B (yellow) binds to the *ERG11* distal promoter region (PDR), aiding in transcriptional activation of *ERG11*. Upc2A (green) binds to the *ERG11* proximal promoter region (PPR) and is the commonly known transcription factor for *ERG11*. Hap1A is not depicted or control expression of *ERG11* because it is not expressed, as determined by our data. Under hypoxic conditions, Hap1A (blue) is induced and highly expressed where it prefers to bind to the PDR of *ERG11* to repress *ERG11* and to prevent binding of Hap1B in the PDR. Hap1B binds to the PPR region and could help in repression. (**B**) Under aerobic conditions with fluconazole treatment, Hap1B (yellow) binds to the *ERG3* distal promoter region (PDR), aiding Upc2A in transcriptional activation of *ERG3*. Upc2A (green) is known to bind to the *ERG3* proximal promoter region and a known transcription factor for *ERG3*. Again, Hap1A is not shown to control the expression of *ERG3* because it is not expressed. Under hypoxic conditions, Hap1A is induced and highly expressed where it prefers to bind the PDR. As indicated, both Hap1A and Hap1B bind to the PPR regions of *ERG3* and could help in repression. (**C**) Based on hypoxic gene expression data, Hap1A and Hap1B equally contribute to the repression of *ERG2*. Overall, these models indicate utilization of three zinc cluster transcription factors for direct and distinct promoter control of *ERG* genes in response to azole treatment and hypoxic conditions. Upc2A was not depicted in the hypoxic diagrams since it is unclear if Upc2A is present or absent at the repressed *ERG* genes. However, we would expect that Upc2A is the main transcriptional activator when Hap1A and Hap1B are not present. For panels A–C, arrows represent activation, while bars indicate inhibition. The dotted lines and question marks denote unknown interactions or regulatory mechanisms.

In this study, the roles of the *C. glabrata* zinc cluster transcription factor ohnologs, Hap1A and Hap1B, were investigated in response to azole drug treatment and hypoxic conditions. Our data suggest that Hap1B functions similarly to *S. cerevisiae* Hap1 under aerobic conditions, regulating the conserved genes *CYC1* and *ERG3* under untreated conditions. Additionally, we found that loss of *HAP1B*, but not *HAP1A*, impacts azole susceptibility due to the inability to adequately induce *ERG* genes under azole drug treatment and maintain ergosterol homeostasis. Furthermore, we discovered that *HAP1A* transcript is upregulated, and protein levels are specifically detected in response to hypoxia, allowing it to function as a repressor of *ERG* genes. Overall, our study revealed that *C. glabrata* utilizes Hap1A and Hap1B to control gene expression and mediate proper ergosterol homeostasis in response to both azole drug treatment and hypoxic conditions (see model Fig. 12A and B).

Even though phylogenetic and syntenic analyses indicate that *S. cerevisiae* Hap1 is orthologous to *C. glabrata* Hap1A, we determined that *C. glabrata* Hap1B, but not Hap1A, alters azole susceptibility. Although Upc2A is the major transcription factor associated with azole-mediated induction of *ERG* genes, our study provides new insights into an additional transcriptional regulator besides Upc2A that is needed for azole-induced expression of *ERG* genes. Additional genetic and biochemical studies will be needed to determine the mechanism by which Hap1B and Upc2A operate together in response to azole drugs. Nonetheless, we speculate that Hap1B could mediate either a direct or indirect cooperative event that assists Upc2A in fully inducing ergosterol genes (see model, Fig. 12A and B). Additionally, in *S. cerevisiae*, deleting both Upc2 and its paralog Ecm22 further alters azole drug susceptibility, resistance to amphotericin B, and *ERG* gene expression (42, 43, 45, 63). Thus, Upc2A and Hap1B may be operating in an analogous manner. However, there exists a distinct possibility that other yet-to-be identified zinc cluster transcription factors could be involved in regulating *ERG* gene expression. Identifying additional transcription factors besides Hap1B and Upc2A will be important to fully understand what contributes to azole susceptibility and/or clinical drug resistance.

In contrast to Hap1B, Hap1A protein levels were nearly undetectable under aerobic and/or azole-treated conditions, with significant induction observed only under hypoxic conditions. This explains why the *HAP1A* deletion strain lacks an azole hypersensitive phenotype or any alteration in *ERG* gene expression. Based on our data, Hap1A protein levels are likely being regulated by an unknown post-transcriptional mechanism. Although we have not identified the regulatory mechanism governing Hap1A protein levels, we suspect that it is degraded via a specific ubiquitin ligase. Hap1A may also be regulated in a manner similar to human HIF-1α (65, 66). To our knowledge, Hap1A represents the first identified hypoxia-induced zinc cluster transcription factor and understanding the precise mechanism of protein degradation would be of interest.

Hap1A was previously named Mar1 (Multiple Azole Resistance 1) because deletion of Hap1A in the *C. glabrata* KUE100 strain was shown to alter azole susceptibility when treated with high concentrations of azoles, we have not been able to confirm this with our studies using the *C. glabrata* 2001 strain (34, 39). Currently, the reason behind these discrepancies is unclear, but there could be differences in *C. glabrata* strains or conditions where Hap1A is expressed at higher levels than what we have observed. However, the findings by Gale et al., utilizing a *C. glabrata* BG14 strain and employing a *Hermes* transposon approach to screen for fluconazole susceptibility, provided support for our observations (67). In their study, they identified several genes that when disrupted, altered azole drug susceptibility, including Hap1B but not Hap1A (67). More studies will be needed to completely understand the role of Hap1A in azole susceptibility, if any, and how it is regulated at the transcriptional and post-transcriptional level. Nonetheless, our results are clear and consistent where Hap1A and Hap1B can play hypoxia-specific roles in repressing *ERG* genes (see model, Fig. 12A through C). We suspect that Hap1A and/or Hap1B, similar to Hap1 in *S. cerevisiae*, operates with a corepressor complex to repress *ERG* genes (57, 63, 64). Hypoxia-induced expression of Hap1A and the growth defect observed in the *hap1A*Δ and *hap1A*Δ*hap1B*Δ strains under hypoxic conditions highlight the importance of these transcription factors in metabolic adaptation and survival in oxygen-limited environments. In addition, it is likely Hap1A hypoxia-specific induction plays additional roles for *C. glabrata* to survive and propagate under low oxygen while within the humans.

Overall, this study expands our understanding of the transcriptional regulation of ergosterol biosynthesis in *C. glabrata*. This is significant because targeting ergosterol and/or enzymes involved in ergosterol biosynthesis have yielded highly useful and effective antifungals (64, 68, 69). Thus, studies focused on the regulatory mechanisms of this pathway could lead to the development of targeted antifungal therapies and help in overcoming the challenge of azole resistance in clinical settings. Because zinc cluster transcription factors are unique to fungi and not found in humans (32), there could be an opportunity to explore them as drug targets. Overall, our findings reveal a novel regulatory mechanism where Hap1A and Hap1B are differentially employed by *C. glabrata* to manage ergosterol biosynthesis and maintain membrane integrity under varying environmental conditions. Our findings provide some of the first insights into the functional role of two zinc cluster transcription factors. We suspect that further studies on these and similar factors will enhance our understanding of the pathophysiology and drug resistance mechanisms of *C. glabrata*.

## MATERIALS AND METHODS

### Plasmids and yeast strains

All plasmids and yeast strains are described in Table S3 and Table S4. The S288C BY4741 *S. cerevisiae* strain was obtained from Open Biosystems. The S288C strain containing the *HAP1-Ty1* sequence was corrected with a wild-type copy of *HAP1* (FY2609), and the *HAP1* deletion strain (FY2611) was kindly provided to us by Dr. Fred Winston, Department of Genetics, Harvard Medical School (40). *C. glabrata* 2001 (CBS138, ATCC 2001) and *C. glabrata* 989 (ATCC 200989) were acquired from the American Type Culture Collection (70). For Hap1B complementation assays, a genomic fragment containing the *HAP1B* promoter, 5′ UTR, open reading frame (ORF), and 3′ UTR was PCR-amplified and cloned into the pGRB2.0 plasmid (Addgene) (71) using restriction enzymes BamHI and SacII. For endogenous C-terminal epitope tagging, a 3×FLAG-NatMX cassette was PCR-amplified from pYC46 plasmid (Addgene) and inserted at the C-terminus of *HAP1B* and *HAP1A* (72, 73). All *C. glabrata* strains were created using the CRISPR-Cas9 RNP system as previously described (72). Briefly, for generating deletion strains, two CRISPR gRNAs were designed near the 5′ and 3′ ORFs of the gene of interest. Drug-resistant selection markers were PCR-amplified using Ultramer DNA Oligos (IDT) from pAG32-HPHMX6 (hygromycin) or pAG25-NATMX6 (nourseothricin). For 3×FLAG epitope tagging, one CRISPR gRNA was designed in the 3′ UTR of the gene of interest. Cells were then electroporated with the CRISPR-RNP mix and the drug resistance cassette.

### Serial-dilution spot and liquid growth assay

For serial-dilution spot assays, yeast strains were grown to saturation overnight in synthetic complete (SC) media at 30°C. Cells were diluted to OD_600_ of 0.1 and allowed to grow to exponential phase with continuous shaking at 30°C. Each strain was then spotted in fivefold dilution starting at an OD_600_ of 0.01 on untreated SC agar plates or plates containing 16 µg/mL fluconazole (Cayman) for *S. cerevisiae* and 32 µg/mL fluconazole (Cayman) for *C. glabrata*. Plates were grown at 30°C for 2 days. For liquid growth assay, the yeast strains were inoculated in SC media and grown to saturation overnight. The cultures were then diluted to an OD_600_ of 0.1 and grown to log phase with shaking at 30°C. Upon reaching log phase, the strains were diluted to an OD_600_ of 0.001 in a 96-well round bottom plate containing 100 µL of SC media with and without 16 µg/mL fluconazole for *S. cerevisiae* and 32 µg/mL fluconazole for *C. glabrata*. Fluconazole, a triazole antifungal commonly used to treat *Candida* infections, was dissolved in sterile purified water for use in these studies. Cells were grown in liquid culture for 50 h with shaking at 30°C, and the OD_600_ was measured every 15 min using a Bio-Tek Synergy four multimode plate reader. For spot assays under hypoxia, YPD plates were placed inside the BD Gaspak EZ anaerobe gas generating pouch system with indicator (BD 260683) after spotting and incubated for up to 7 days. Hypoxic cell collection for qRT-PCR, Western blot, and ChIP assays was performed by growing the indicated yeast strains in YPD media for 8 h using the BD GasPak EZ anaerobe gas-generating pouch system (BD 260683). Cells were immediately spun down for 1 min and flash frozen to maintain the hypoxic state.

### Phylogenetic analysis

For the phylogenetic analysis, the multiple sequence alignment for the full *HAP1* gene family was downloaded from the Yeast Gene Order Browser (http://ygob.ucd.ie/) (50). This file included homologous genes for 19 species: 11 united by a whole genome duplication event in their last common ancestor (*S. cerevisiae, S. kudriavzevii, S. mikatae, C. glabrata, Kazachstania africana, Kazachstania naganishii, Naumovozyma castellii, Naumovozyma dairenensis, Tetrapisispora blattae, Tetrapisispora phaffii*, and *Vanderwaltozyma polyspora*) and eight outgroups (*Eremothecium cymbalariae, Eremothecium gossypii, Kluyveromyces lactis, Lachancea kluyveri, Lachancea thermotolerans, Lachancea waltii, Torulaspora delbrueckii,* and *Zygosaccharomyces rouxii*). Additionally, we identified Hap1 homologs in *Nakaseomyces bacillisporus* and *Nakaseomyces delphensis*, two species related to *C. glabrata,* by querying the Hap1 sequence against both genomes in JGI Mycocosm using the built in BLAST tool (74). Combined, all sequences were realigned using Muscle5 v 5.1 using default settings (75). The aligned sequences were then used to generate a maximum-likelihood phylogenetic tree with IQ-TREE version 2.2.0, using the built-in ModelFinder to determine the best-fit nucleic acid substitution model and 1,000 ultrafast bootstrap replicates (76). The tree was visualized using ETE v3 (77).

### Quantitative real-time PCR analysis

RNA was isolated from *C. glabrata* strains grown in SC or YPD treated with or without 64 µg/mL fluconazole using standard acid phenol purification method, and 1 µg RNA was reverse transcribed to cDNA using the All-in-One 5× RT Mastermix kit (ABM). Gene expression primers were designed using Primer Express 3.0 software and are listed in Table S5. Quantitative real-time polymerase chain reaction (qRT-PCR) values are indicated in Tables S7 and S9. At least three biological replicates, including three technical replicates, were performed for all samples. Data were analyzed by the comparative *C_T_* method (2*^–^*^ΔΔ^*^CT^*) where *RDN18* (18S rRNA) was used as an internal control. All samples were normalized to untreated untagged wild-type strain. GraphPad Prism version 9.5.1 was used to determine the unpaired *t*-test for determining statistical significance.

### Yeast extraction and Western blot analysis

The indicated *C. glabrata* strains were grown in SC or YPD media under aerobic conditions with or without 64 µg/mL of azoles or hypoxic conditions. Yeast whole cell extraction and Western blot analysis to detect Hap1A-3×FLAG, Hap1B-3×FLAG, and histone H3 were performed as previously described (78). The monoclonal FLAG M2 mouse antibody (F1804, Sigma-Aldrich) was used at a 1:5,000 dilution to detect Hap1A-3×FLAG and Hap1B-3×FLAG. as previously described (57). The histone H3 rabbit polyclonal antibody (PRF&L) was used at a 1:100,000 dilution as previously described (72). HRP-conjugated anti-rabbit or anti-mouse IgG was used as secondary antibody.

### Chromatin immunoprecipitation

Chromatin immunoprecipitation was performed using ZipChIP as previously described (79). Briefly, 50 mL cultures of indicated yeast strains were grown to exponential phase (OD_600_ of 0.6) in SC or YPD media with or without shaking at 30°C under aerobic or 8 h of hypoxic condition, respectively. Cells grown in SC media under aerobic condition were treated with 64 µg/mL fluconazole (Cayman) for 3 h and collected. Cells were then formaldehyde cross-linked for 15 min and harvested as previously described (79). The cells were lysed by bead-beating with glass beads, and lysate was separated from beads. Upon separation, cell lysates were transferred to Diagenode Bioruptor Pico microtubes and sonicated with a Diagenode Bioruptor Pico at the high frequency setting for 30 s ON and 30 s OFF for 20 cycles. After sonication, cell lysates were pre-cleared with 5 µL of unbound protein G magnetic beads (10004D, Invitrogen) for 30 min with rotation at 4°C. Then, 300 µL of precleared lysate was immunoprecipitated with 10 µL of protein G- magnetic beads (10004D, Invitrogen) conjugated to 1 µL of M2 FLAG antibody (F1804, Sigma-Aldrich). Probe and primer sets used for qPCR analysis are described in Table S6, and qPCR values are indicated in Tables S8 and S10.

### Ergosterol extraction

Ergosterol was extracted from indicated strains as previously described (58, 80). Cultures were grown overnight in SC minimal media. Saturated cultures were back diluted to OD_600_ of 0.1 and were grown at 30°C to exponential phase (OD_600_ of 0.6), with or without 64 µg/mL fluconazole treatment. Sterols were extracted from yeast using 4 M potassium hydroxide in 70% (vol/vol) ethanol at 85°C for 1 h. After extraction, nonpolar lipids were separated by washing with methanol twice. Nonpolar sterols were crystallized after evaporating the n-hexane and dissolved in 100% methanol. Samples were analyzed by HPLC using a C18 column with a flow rate of 1 mL/min of 100% methanol. Ergosterol was detected at 280 nm, and cholesterol, used as an internal control for extraction, was detected at 210 nm.

## SUPPLEMENTAL MATERIAL

Supplemental Material msphere.00524-24-s0001.pdf Figures S1–S6 and Tables S1–S10.

ASM does not own the copyrights to Supplemental Material that may be linked to, or accessed through, an article. The authors have granted ASM a non-exclusive, world-wide license to publish the Supplemental Material files. Please contact the corresponding author directly for reuse.
