# Supplementary material for: Candida glabrata maintains two HAP1 ohnologs, HAP1A and HAP1B, for distinct roles in ergosterol gene regulation to mediate sterol homeostasis under azole and hypoxic conditions
Source: mSphere. 2024 Oct 23;9(11):e00524-24. doi: 10.1128/msphere.00524-24 (PMC11580460; doi:10.1128/msphere.00524-24)
Supplement: Supplemental Material — Figures S1–S6 and Tables S1–S10. [file msphere.00524-24-s0001.pdf]

Supplemental Material – Tables and Figures

Supplemental Table S1 – Quantification of Yeast Liquid Growth Assays from Figure 1C and D				
(-) Fluconazole				
**Strain**	**BY WT**	**BY*hap1Δ***	**FY WT**	**FY*hap1Δ***
Doubling Time (mins)	112.3 ± 11.7	108.6 ± 9.8	110.9 ± 3.8	109.7 ± 4.2
Time (mins) OD_{600} = 0.5	860 ± 8.7	870 ± 25.9	875 ± 17.3	885 ± 15
(+) Fluconazole				
**Strain**	**BY WT**	**BY*hap1Δ***	**FY WT**	**FY*hap1Δ***
Doubling Time (mins)	225.7 ± 17.7	186.8 ± 9.7	141.2 ± 6.5	201.3 ± 4.1
Time (mins) OD_{600} = 0.5	1775.3 ± 53.2	1340 ± 70.9	1020 ± 0	1340 ± 48.2

Table S1. BY4741 and FY2611 WT and *hap1Δ* strains liquid growth calculations with and without azole treatment. The doubling times and the time required to reach an OD_{600} of 0.5 for indicated strains as determined by liquid growth assays in synthetic complete (SC) media with and without 16 µg/mL fluconazole. Values are expressed as the mean ± SD (standard deviation) and are based on three biological replicates.

Supplemental Table S2 – Quantification of Yeast Liquid Growth Assays from Figure 3B and C			
**(-) Fluconazole**			
**Strain**	**WT**	***hap1BΔ***	***hap1AΔ***
Doubling Time (mins)	117.9 ± 6.7	124.7 ± 1.9	114.7 ± 0.08
Time (mins) OD_{600} = 0.5	670 ± 22.9	705 ± 15	660 ± 0
**(+) Fluconazole**			
**Strain**	**WT**	***hap1BΔ***	***hap1AΔ***
Doubling Time (mins)	154.6 ± 4.1	173.2 ± 2.7	142.3 ± 10.9
Time (mins) OD_{600} = 0.5	1065 ± 39.7	1380 ± 26	1030 ± 22.9

Table S2. Liquid growth calculations of *Cg*2001 WT, *hap1BΔ*, and *hap1BΔ* strains treated with and without azoles. Quantified doubling times and the time required to reach an OD_{600} of 0.5 for *Cg*2001 WT, *hap1BΔ* and *hap1AΔ* strains when grown in synthetic complete (SC) media with and without 32 µg/mL fluconazole. Values are expressed as the mean ± SD (standard deviation) and are based on three biological replicates

Supplemental Figure S1

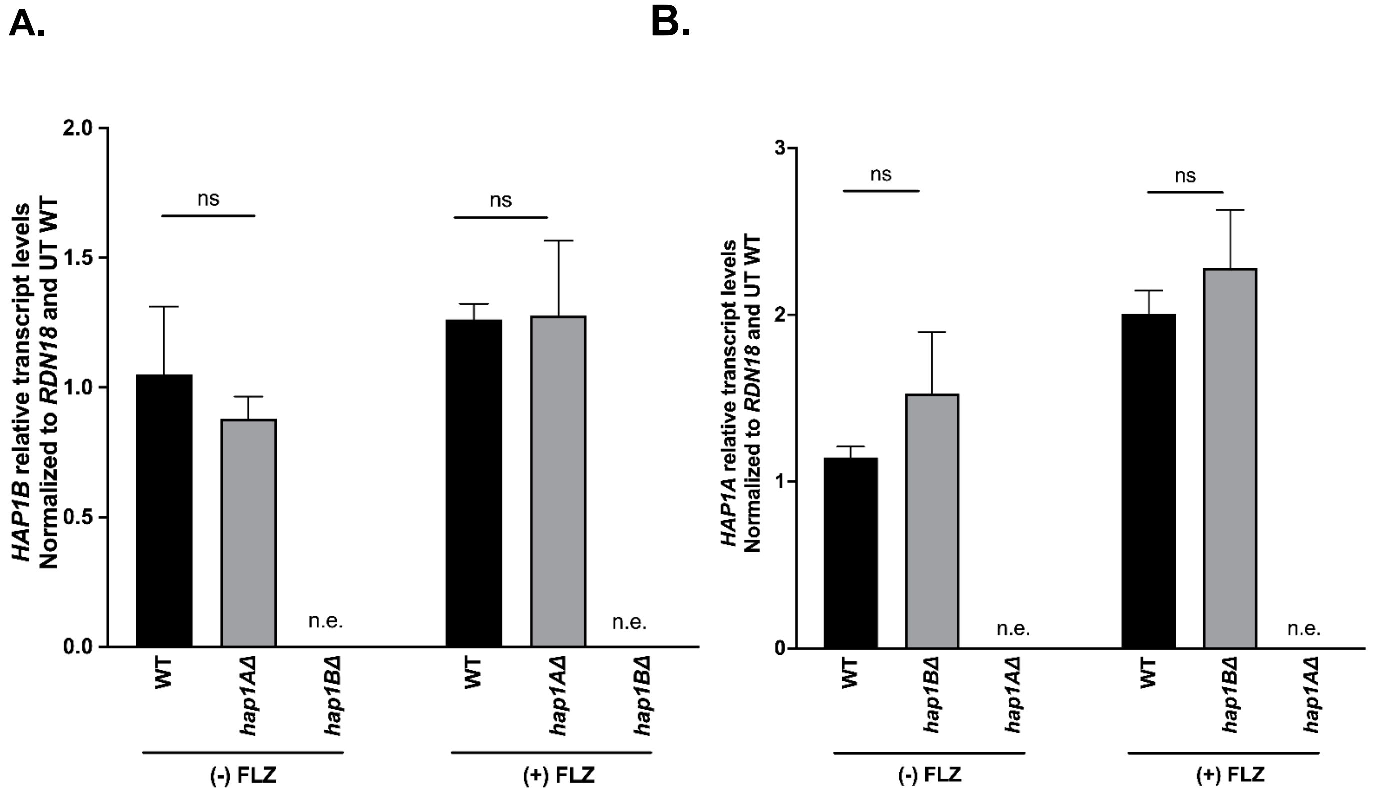

Fig S1. Transcript levels in *Cg*2001 WT, *hap1AΔ*, and/or *hap1BΔ* strains with or without azole treatment. **(A-B)** Transcript levels of *HAP1B* and *HAP1A* were determined from the indicated cells treated with and without 64 μg/mL fluconazole for 3 hr by qRT-PCR analysis. Gene expression analysis was set relative to untreated WT samples and expression was normalized to *RDN18* levels. Data were analyzed from four biological replicates with three technical replicates. Statistics were determined using the GraphPad Prism Student t-test, version 9.5.1: Error bars represent standard deviation (SD); Not significant = ns; not expressed = n.e.; Mean RQ , Mean RQ = 1.025 (WT, (-) FLZ), 0.82 (*hap1AΔ*, (-) FLZ), 0 (*hap1BΔ*, (-) FLZ), 1.25 (WT, (+) FLZ), 1.25 (*hap1AΔ*, (+) FLZ), 0 (*hap1BΔ*, (+) FLZ); 1.14 (WT, (-) FLZ), 1.52 (*hap1BΔ*, (-) FLZ), 0 (*hap1AΔ*, (-) FLZ), 2 (WT, (+) FLZ), 2.27 (*hap1BΔ*, (+) FLZ), 0 (*hap1AΔ*, (+) FLZ)

Supplemental Figure S2

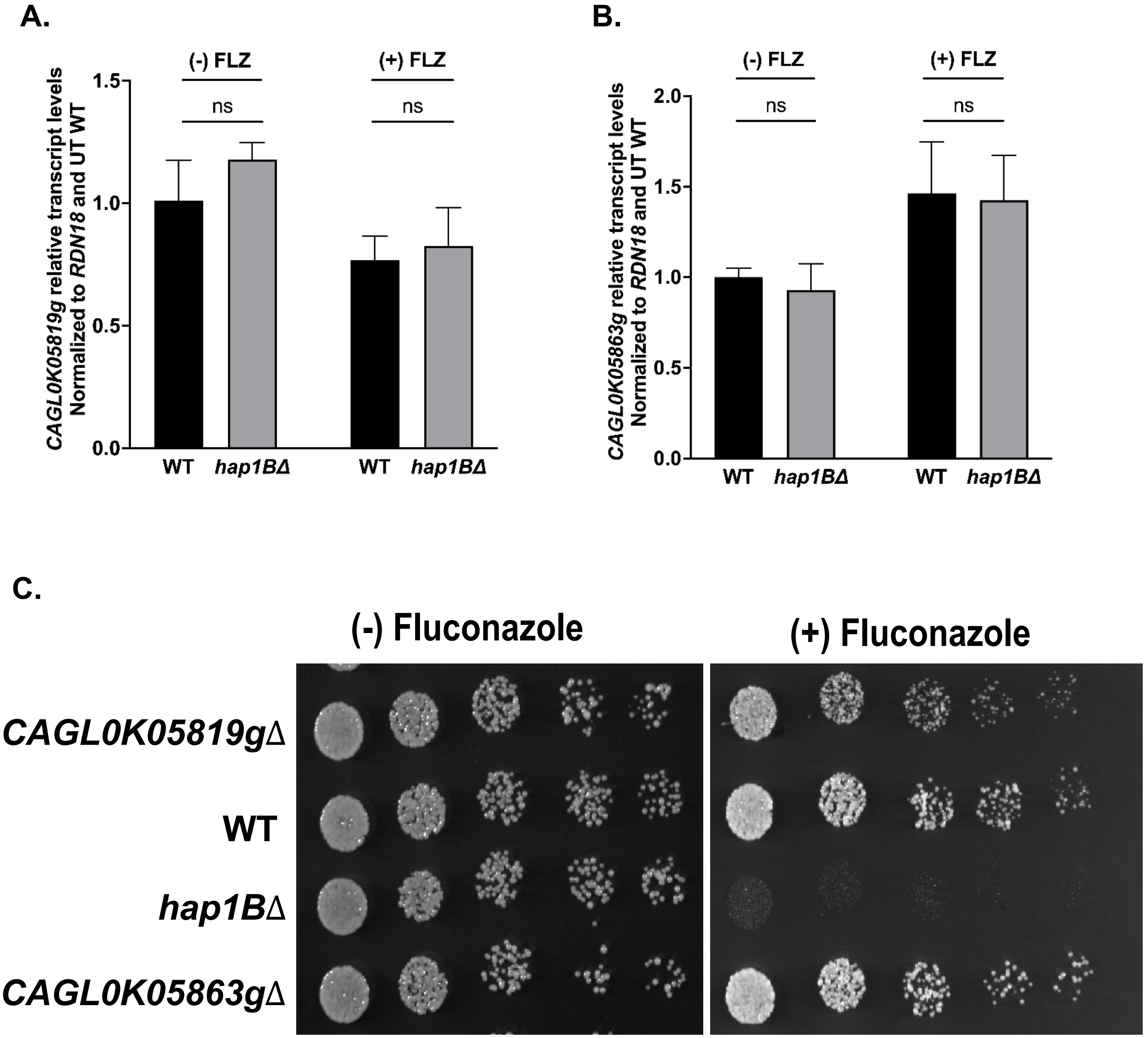

Fig S2. Transcript level and azole susceptibility of genes located upstream (*CAGL0K05819g*) and downstream (*CAGL0K05863g*) of *HAP1B*. **(A and B)** Transcript levels of *CAGL0K05819g* and *CAGL0K05863g* treated with and without 64 µg/mL fluconazole for 3 hr were determine by qRT-PCR analysis. Transcript levels were set relative to untreated WT samples and normalized to *RDN18* levels. Data were analyzed from three biological replicates with three technical replicates. Statistics were determined using the GraphPad Prism Student t-test, version 9.5.1. Error bars represent standard deviation (SD) and not significant = ns; Mean RQ= 0.99 (WT, (-) FLZ), 1.17 (*hap1BΔ*, (-) FLZ), 0.76 (WT, (+) FLZ), 0.83 (*hap1BΔ*, (+) FLZ); 0.99 (WT, (-) FLZ), 0.92 (*hap1BΔ*, (-) FLZ), 1.4 (WT, (+) FLZ), 1.4 (*hap1BΔ*, (+) FLZ) **(C)** Azole susceptibility of the *Cg*2001 WT, *hap1BΔ*, *CAGL0K05819gΔ* and *CAGL0K05863gΔ* strains. Five-fold serial dilution assays of indicated strains grown on SC plates with and without 32 µg/mL fluconazole and incubated at 30°C for 48 hours.

Supplemental Figure S3

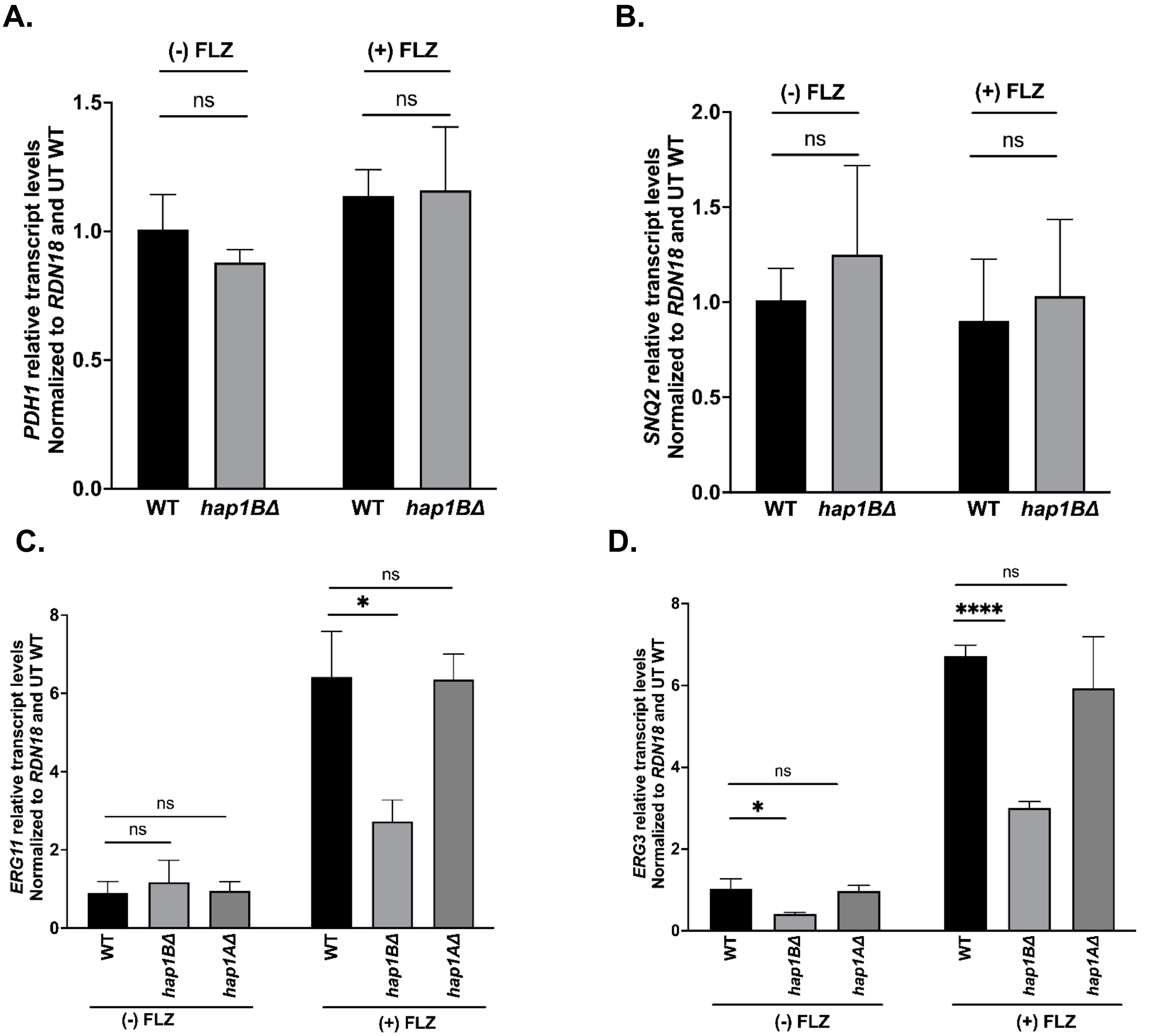

Fig S3. Transcript levels in *Cg*2001 WT, *hap1B*Δ, and/or *hap1AΔ* strains with or without azole treatment. **(A-B)** Transcript levels of drug transporters *PDH1* and *SNQ1* were determined from the indicated cells treated with and without 64 µg/mL fluconazole for 3 hr by qRT-PCR analysis. Gene expression analysis was set relative to untreated WT samples and expression was normalized to *RDN18* levels. Data were analyzed from four biological replicates with three technical replicates. Statistics were determined using the GraphPad Prism Student t-test, version 9.5.1: Error bars represent standard deviation (SD); Not significant = ns; Mean RQ = 1.00 (WT, (-) FLZ), 0.87 (*hap1BΔ*, (-) FLZ), 1.12 (WT, (+) FLZ), 1.15 (*hap1BΔ* (+) FLZ); 1.007 (WT, (-) FLZ), 1.24(*hap1BΔ*, (-) FLZ), 0.89 (WT, (+) FLZ), 1.07 (*hap1BΔ*, (+) FLZ); **(C-D)** Transcript levels of *ERG11* and *ERG3* were determined from indicated cells treated with and without 64 µg/ml fluconazole for 3 hr by qRT-PCR analysis. Gene expression analysis was set relative to untreated WT samples and expression was normalized to *RDN18* levels. Data were analyzed from three biological replicates with three technical replicates. Statistics were determined using the GraphPad Prism Student t-test, version 9.5.1: Error bars represent standard deviation (SD), not significant = ns, Mean RQ = 0.9 (WT, (-) FLZ), 1.17 (*hap1BΔ*, (-) FLZ), 0.95 (*hap1AΔ,* (-) FLZ), 6.43 (WT, (+) FLZ), 2.72 (*hap1BΔ,* (+) FLZ), 6.35 (*zcfΔ*, (+) FLZ); 1.05 WT, (-) FLZ), 0.40 (*hap1BΔ*, (-) FLZ), 0.96 (*hap1AΔ*, (-) FLZ), 6.718 (WT, (+) FLZ), 3 (*hap1BΔ*, (+) FLZ), 5.93 (*hap1AΔ*, (+) FLZ); P > 0.05; *, P < 0.05; *, ****, P < 0.0001.

Supplemental Figure S4

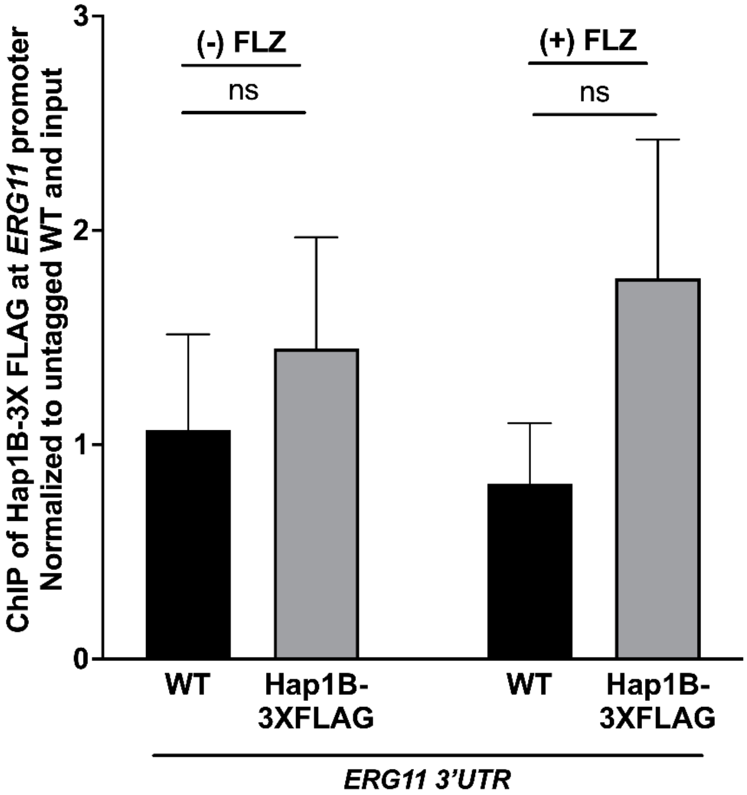

Fig S4. Chromatin immunoprecipitation analysis of Hap1B at *ERG11* 3'*UTR* upon fluconazole treatment. *Cg*2001 WT and Hap1B-3XFLAG strains were treated with and without 64 μg/mL fluconazole. ChIP analysis was set relative to untagged WT samples and normalized to input. Data were analyzed from three biological replicates with three technical replicates. Statistics were determined using the GraphPad Prism Student t-test, version 9.5.1: Error bars represent standard deviation (SD), not significant = ns, P > 0.05, Mean RQ = 1.06 (WT, (-) FLZ), 1.43 (Hap1B-3XFLAG, (-) FLZ), 0.81 (WT, (+) FLZ), 1.7 (Hap1B-3XFLAG, (+) FLZ).

Supplemental Figure S5

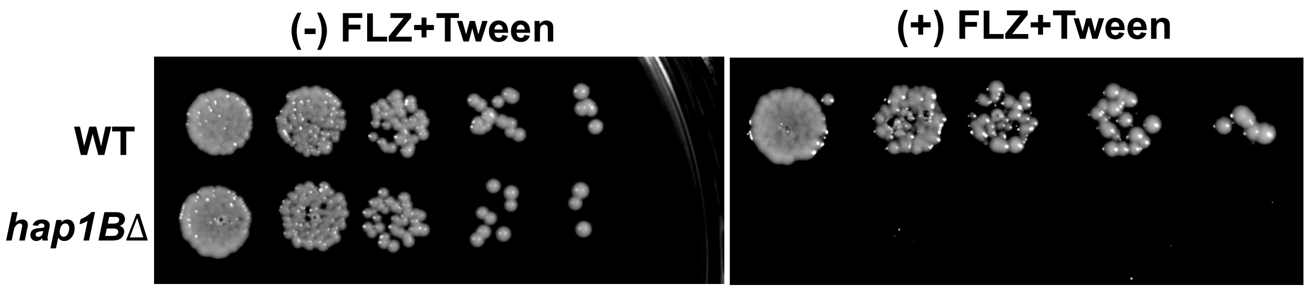

Fig S5. Fivefold serial dilution assays of WT and *hap1BΔ* strain grown on agar plates. Dilution assays of indicated strains spotted on SC plates with and without 32 µg/mL fluconazole plus 2% Tween 80-ethanol solution. Agar plates were incubated at 30°C for 48 hours.

Supplemental Figure S6

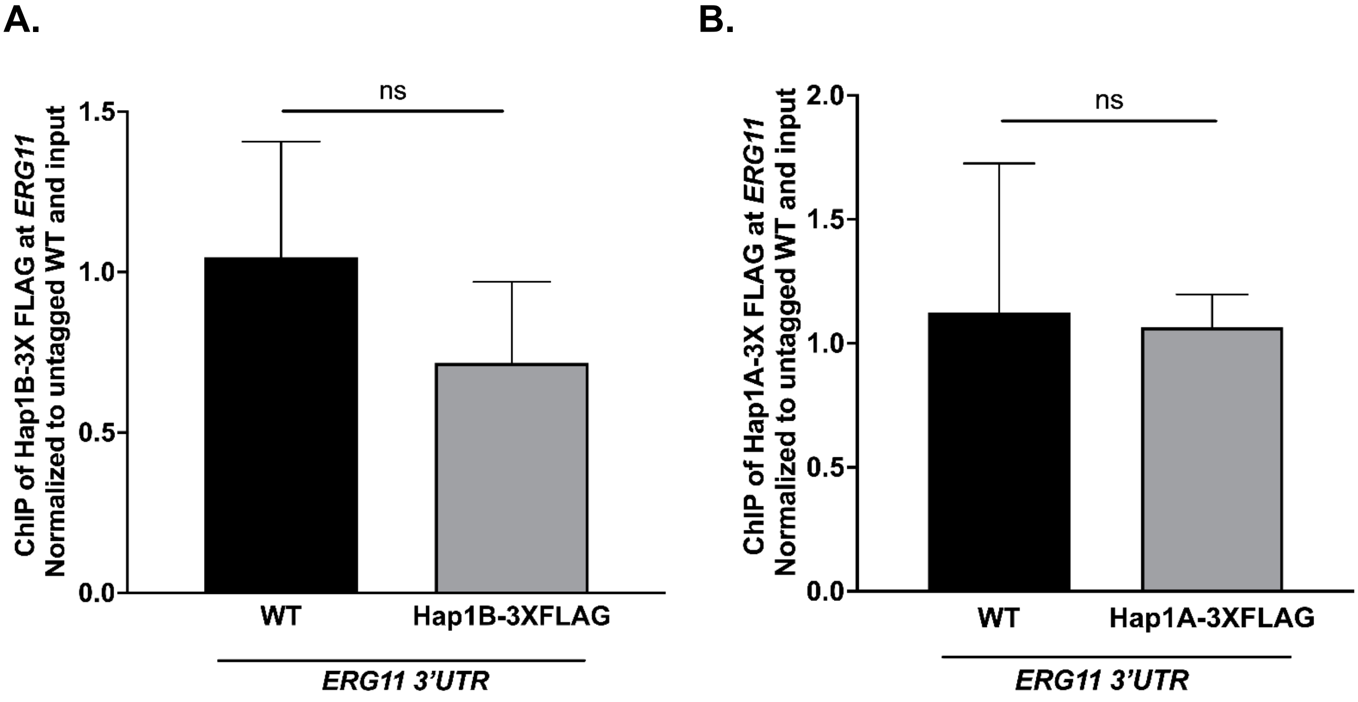

Fig S6. Chromatin immunoprecipitation analysis of Hap1B and Hap1A at *ERG11* 3'*UTR*. *Cg*2001 WT, Hap1B-3XFLAG and Hap1A-3XFLAG strains were grown under hypoxic conditions **(A-B)**. ChIP analysis was set relative to untagged WT samples and normalized to input. Data were analyzed from three biological replicates with three technical replicates. Statistics were determined using the GraphPad Prism Student t-test, version 9.5.1: Error bars represent standard deviation (SD), not significant = ns, P > 0.05, **(A)** Mean RQ = 1.04 (WT) and 0.713 (Hap1B-3XFLAG); **(B)** Mean RQ = 1.12 (WT) and 1.06 (Hap1B-3XFLAG)

Supplemental Table S3: Yeast Strains and Genotype			
**Yeast name**	**Genotype**	**Reference**	**Strain**
S. cerevisiae BY4741	*MATα his3Δ leu2Δ0 LYS2 met15Δ0 ura3Δ0*	Open Biosystems	BY4741 WT
SDBY 1638	*MATα his3Δ leu2Δ0 LYS2 met15Δ0 ura3Δ0 hap1Δ::NatMX*	This study	BY4741 *hap1Δ*
S. cerevisiae FY2609	*MATa ura3Δ his3Δ200 leu2Δ lys2-128δ HAP1*	Kindly provided by Fred Winston (1)	FY2609 WT
FY2611	*MATa ura3Δ his3Δ200 leu2Δ0 lys2-128δ hap1Δ::KanMX*	Kindly provided by Fred Winston (1)	FY2609 *hap1Δ*
ATCC 2001	*C. glabrata* prototrophic reference strain	www.atcc.org	*Cg*2001 WT
SDBY 1639	*hap1BΔ::NatMX*	This study	*Cg*2001 *hap1BΔ*
SDBY1640	*hap1AΔ::NatMX*	This study	*Cg*2001 *hap1AΔ*
SDBY 1641	*hap1AΔ::HphMXhap1BΔ::NatMX*	This study	*Cg*2001 *hap1AΔhap1BΔ*
SDBY 1642	*aus1Δ::NatMX*	This study	*Cg*2001 *aus1Δ*
SDBY 1643	*aus1Δ::NatMX hap1BΔ::HphMX*	This study	*Cg*2001 *hap1BΔaus1Δ*
ATCC 200989	*his3Δ trp1Δ ura3Δ*	www.atcc.org	*Cg*989 WT
SDBY 1644	*his3Δ trp1Δ ura3Δ hap1BΔ::NatMX*	This study	*Cg989 hap1BΔ*
SDBY 1645	*HAP1B-3XFLAG::NatMX*	This study	*Cg*2001 *HAP1B-3XFLAG*
SDBY 1646	*HAP1A-3XFLAG::NatMX*	This study	*Cg*2001 *HAP1A-3XFLAG*

Supplemental Table S4: Plasmids				
**Plasmid**	**Insert Gene**	**Promoter**	**3'UTR**	**Source**
pGRB2.0	None	None	None	(2)
pGRB2.0	*HAP1B* (4119bp ORF)	*HAP1B* (886bp)	*HAP1B* (359bp)	This study

**Supplemental Table S5: Primers for qRT-PCR Expression Analysis**	
**Primer Name**	**Sequence**
RDN18-001F	ACGGAGCCAGCGAGTCTAAC
RDN18-002R	CGACGGAGTTTCACAAGATTACC
HAP1B-001F	AGGGTGCCGTTGAGCTGTAC
HAP1B-002R	GGCGGGACTTATCACACTTGA
HAP1A-001F	ACTCTCCTGTTGGATTGAGTTC
HAP1A-002R	AATATGGTTGACGACATGGCTA
CYC1-001F	CACGGTATCTTCGGTAGAAAGTCA
CYC1-002R	ATGTTGGCGTCGGTGTAGGA
CDR1-001F	GTCTATGGAAGGTGCCGTCAA
CDR1-002R	TGAACCAGGTCTACCTAGCACAAC
PDR1-001F	TCGGCGAGGGTAAATTCAAC
PDR1-002R	CAACTGCGTTTGATTCCTTAAGC
ERG11-001F	GGGTCCAAAGGGTCACGAA
ERG11-002R	GCAGCTTCAGCGGAAACATC
ERG3-001F	TGGGAGCACCACGGTCTAAG
ERG3-002R	CAGTCGGTGAAGAAGATGAAAGTG
ERG2-001F	GGATCAAAACTCCTTCGCACTAG
ERG2-002R	AACCGAATGGCAACATGCAT
ERG5-001F	GTCACCGCCGCTTTGG
ERG5-002R	CCGTACCAGGTCTTGGTGAAA

**Supplemental Table S6: Probe and Primer Sets for Chromatin Immunoprecipitation Analysis.**		
**Promoter**	**Probe & Primers**	**Sequence 5'-3'**
ERG11 Promoter (PDR)	Probe	5’-/56-FAM/CCTTGTTCC/ZEN/AACTACAATCGAGTGAGCT/3IABkFQ/-3’
	Primer	5’-CGAATACGAGGCCATTTGTAAAC-3’
	Primer	5’-CTGTGCTCCCATCTCACTATAAC-3’
ERG11 Promoter (PPR)	Probe	5’-/56-FAM/TGCTCAGCA/ZEN/CAGTGA/3IABkFQ/-3’
	Primer	5’-ATCGGTCCATCTCTGTTTCTTT-3’
	Primer	5’-CGAGATGAGTATACGGGTTCTTC-3’
ERG11 3’*UTR*	Probe	5’-/56-FAM/CGGCATGAC/ZEN/TTAAGCTGGTTGTTTCG/3IABkFQ/-3’
	Primer	5’-ACGGGATATACGCTGATTCATT-3’
	Primer	5’-AGCAGCAAAGCCCTCTAAA-3’
ERG3 Promoter	Probe	5’-/56-FAM/TGAACGGAG/ZEN/ATAAGGTATACGACCGT/3IABkFQ/-3’

(PDR)		
	Primer	5'-TGAACGGAGATAAGGTATACGACCGT-3'
	Primer	5'-GCAAAGAGCACACTCGTATAGT-3'
ERG3 Promoter (PPR)	Probe	5'-/56-FAM/CATAATTGC/ZEN/CCAGTGCAGCCATCG/3IABkFQ/-3'
	Primer	5'-AGAGCCTTGAGGAGAGATGA-3'
	Primer	5'-GTTCTCGTAGTTCCCACGTATAG-3'

Supplemental Table S7 – qRT-PCR Expression Analysis

Gene	Strain	Condition	Mean RQ	St Dev	n	P-value
**Figure 4A - qRT-PCR Values**						
**Gene**	**Strain**	**Condition**	**Mean RQ**	**St Dev**	**n**	**P-value**
CYC1	2001 WT	Untreated	1.00	0.14	3	n.s.
CYC1	*hap1BΔ*	Untreated	0.47	0.14	3	<0.01
CYC1	*hap1AΔ*	Untreated	1.23	0.24	3	n.s.
**Figure 4B - qRT-PCR Values**						
**Gene**	**Strain**	**Condition**	**Mean RQ**	**St Dev**	**n**	**P-value**
CYC1	2001 WT	(+) fluconazole	1.02	0.26	3	n.s.
CYC1	*hap1BΔ*	(+) fluconazole	0.41	0.16	3	<0.01
CYC1	*hap1AΔ*	(+) fluconazole	1.20	0.42	3	n.s.
**Figure 4C - qRT-PCR Values**						
**Gene**	**Strain**	**Condition**	**Mean RQ**	**St Dev**	**n**	**P-value**
HAP1B	2001 WT	Untreated	1.12	0.12	3	n.s.
HAP1B	2001 WT	(+) fluconazole -3hrs	1.20	0.15	3	n.s.
HAP1B	2001 WT	(+) fluconazole -6hrs	1.33	1.32	3	n.s.
**Figure 4D - qRT-PCR Values**						
**Gene**	**Strain**	**Condition**	**Mean RQ**	**St Dev**	**n**	**P-value**
HAP1A	2001 WT	Untreated	1.09	0.06	4	n.s.
HAP1A	WT	(+) fluconazole -3hrs	1.27	0.20	4	n.s.
HAP1A	WT	(+) fluconazole -6hrs	1.41	0.13	4	n.s.
**Figure 5A - qRT-PCR Values**						
**Gene**	**Strain**	**Condition**	**Mean RQ**	**St Dev**	**n**	**P-value**
CDR1	2001 WT	Untreated	0.83	0.15	3	n.s.
CDR1	2001 WT	(+) fluconazole	5.58	1.31	3	n.s.
CDR1	*hap1BΔ*	Untreated	1.34	0.55	3	n.s.
CDR1	*hap1BΔ*	(+) fluconazole	4.48	0.85	3	n.s.
**Figure 5B - qRT-PCR Values**						
**Gene**	**Strain**	**Condition**	**Mean RQ**	**St Dev**	**n**	**P-value**
PDR1	2001 WT	Untreated	1.04	0.4	3	n.s.

PDR1	2001 WT	(+) fluconazole	2.78	0.29	3	n.s.
PDR1	*hap1BΔ*	Untreated	0.81	0.29	3	n.s.
PDR1	*hap1BΔ*	(+) fluconazole	2.76	0.57	3	n.s.

Figure 5C - qRT-PCR Values

Gene	Strain	Condition	Mean RQ	St Dev	n	P-value
ERG11	2001 WT	Untreated	1.08	0.39	4	n.s.
ERG11	2001 WT	(+) fluconazole	8.01	1.42	4	n.s.
ERG11	*hap1BΔ*	Untreated	0.61	0.13	4	n.s.
ERG11	*hap1BΔ*	(+) fluconazole	3.29	1.20	4	<0.01

Figure 5D - qRT-PCR Values

Gene	Strain	Condition	Mean RQ	St Dev	n	P-value
ERG3	2001 WT	Untreated	1.18	0.37	4	n.s.
ERG3	2001 WT	(+) fluconazole	5.55	0.70	4	n.s.
ERG3	*hap1BΔ*	Untreated	0.58	0.11	4	<0.05
ERG3	*hap1BΔ*	(+) fluconazole	3.05	0.88	4	<0.01

Figure 5E - qRT-PCR Values

Gene	Strain	Condition	Mean RQ	St Dev	n	P-value
ERG2	2001 WT	Untreated	0.88	0.18	3	n.s.
ERG2	2001 WT	(+) fluconazole	16.07	2.89	3	n.s.
ERG2	*hap1BΔ*	Untreated	1.10	0.16	3	n.s.
ERG2	*hap1BΔ*	(+) fluconazole	9.98	2.28	3	<0.05

Figure 5F - qRT-PCR Values

Gene	Strain	Condition	Mean RQ	St Dev	n	P-value
ERG5	2001 WT	Untreated	0.87	0.13	4	n.s.
ERG5	2001 WT	(+) fluconazole	5.48	0.28	4	n.s.
ERG5	*hap1BΔ*	Untreated	0.94	0.22	4	n.s.
ERG5	*hap1BΔ*	(+) fluconazole	3.16	0.96	4	<0.01

Supplemental Table S8 – Chromatin Immunoprecipitation Analysis

Figure 6A: ChIP qPCR Values

Gene	Position	Strain	Condition	Mean RQ	St Dev	n	P-value
ERG11	PDR	2001 WT	Untreated	1.25	0.42	3	n.s.
ERG11	PDR	2001 WT	(+) fluconazole	1.54	0.66	3	<0.001
ERG11	PDR	*HAP1B-3XFLAG*	Untreated	12.68	4.03	3	n.s.
ERG11	PDR	*HAP1B-3XFLAG*	(+) fluconazole	40.33	11.00	3	<0.001

Figure 6B: ChIP qPCR Values

Gene	Position	Strain	Condition	Mean RQ	St Dev	n	P-value
ERG11	PPR	2001 WT	Untreated	1.19	0.31	3	n.s.
ERG11	PPR	2001 WT	(+) fluconazole	0.87	0.26	3	<0.0001
ERG11	PPR	*HAP1B-3XFLAG*	Untreated	5.89	0.65	3	n.s.

ERG11	PPR	*HAP1B-3XFLAG*	(+) fluconazole	11.73	1.48	3	<0.0001

Figure 6C: ChIP qPCR Values							
**Gene**	**Position**	**Strain**	**Condition**	**Mean RQ**	**St Dev**	**n**	**P-value**
ERG3	PDR	2001 WT	Untreated	1.10	0.62	3	n.s.
ERG3	PDR	2001 WT	(+) fluconazole	1.62	0.64	3	<0.05
ERG3	PDR	*HAP1B-3XFLAG*	Untreated	2.45	0.57	3	n.s.
ERG3	PDR	*HAP1B-3XFLAG*	(+) fluconazole	7.98	2.17	3	<0.05

Figure 6D: ChIP qPCR Values							
**Gene**	**Position**	**Strain**	**Condition**	**Mean RQ**	**St Dev**	**n**	**P-value**
ERG3	PPR	2001 WT	Untreated	1.08	0.49	3	n.s.
ERG3	PPR	2001 WT	(+) fluconazole	1.06	0.48	3	n.s.
ERG3	PPR	*HAP1B-3XFLAG*	Untreated	0.77	0.23	3	n.s.
ERG3	PPR	*HAP1B-3XFLAG*	(+) fluconazole	1.07	0.14	3	n.s.

Supplemental Table S9 – qRT-PCR Expression Analysis (hypoxic conditions)

Figure 8C - qRT-PCR Values						
**Gene**	**Strain**	**Condition (Hours in hypoxia)**	**Mean RQ**	**St Dev**	**n**	**P-value**
HAP1A	2001 WT	0	1.01	0.16	3	n.s.
HAP1A	2001 WT	2	3.82	0.88	3	<0.05
HAP1A	2001 WT	4	4.52	0.28	3	<0.05
HAP1A	2001 WT	6	4.68	1.17	3	<0.05
HAP1A	2001 WT	8	5.12	0.70	3	<0.05

Figure 8D - qRT-PCR Values						
**Gene**	**Strain**	**Condition (Hours in hypoxia)**	**Mean RQ**	**St Dev**	**n**	**P-value**
HAP1B	2001 WT	0	0.93	0.15	3	n.s.
HAP1B	2001 WT	2	0.95	0.07	3	n.s.
HAP1B	2001 WT	4	0.85	0.13	3	n.s.
HAP1B	2001 WT	6	0.83	0.18	3	n.s.
HAP1B	2001 WT	8	0.80	0.13	3	n.s.

Figure 9A - qRT-PCR Values						
**Gene**	**Strain**	**Condition**	**Mean RQ**	**St Dev**	**n**	**P-value**
ERG11	2001 WT	Aerobic	1.09	0.32	4	<0.05
ERG11	2001 WT	Hypoxic	0.34	0.13	4	<0.05

Figure 9B - qRT-PCR Values						
**Gene**	**Strain**	**Condition**	**Mean RQ**	**St Dev**	**n**	**P-value**

ERG3	2001 WT	Aerobic	1.03	0.29	3	<0.05
ERG3	2001 WT	Hypoxic	0.24	0.05	3	<0.05

Figure 9C - qRT-PCR Values

Gene	Strain	Condition	Mean RQ	St Dev	n	P-value
ERG2	2001 WT	Aerobic	1.00	0.12	3	<0.05
ERG2	2001 WT	Hypoxic	0.22	0.5	3	<0.05

Figure 9D - qRT-PCR Values

Gene	Strain	Condition	Mean RQ	St Dev	n	P-value
ERG5	2001 WT	Aerobic	1.04	0.33	4	<0.05
ERG5	2001 WT	Hypoxic	0.11	0.01	4	<0.05

Figure 10A - qRT-PCR Values

Gene	Strain	Condition	Mean RQ	St Dev	n	P-value
ERG11	2001 WT	Hypoxic	1.04	0.35	3	n.s.
ERG11	*hap1BΔ*	Hypoxic	1.09	0.14	3	n.s.
ERG11	*hap1AΔ*	Hypoxic	1.66	0.13	3	<0.0001
ERG11	*hap1AΔ hap1BΔ*	Hypoxic	6.34	1.22	3	<0.001

Figure 10B - qRT-PCR Values

Gene	Strain	Condition	Mean RQ	St Dev	n	P-value
ERG3	2001 WT	Hypoxic	1.00	0.02	3	n.s.
ERG3	*hap1BΔ*	Hypoxic	0.89	0.13	3	n.s.
ERG3	*hap1AΔ*	Hypoxic	4.79	1.06	3	<0.001
ERG3	*hap1AΔ hap1BΔ*	Hypoxic	8.25	1.13	3	<0.0001

Figure 10C - qRT-PCR Values

Gene	Strain	Condition	Mean RQ	St Dev	n	P-value
ERG2	2001 WT	Hypoxic	1.02	0.22	3	n.s.
ERG2	*hap1BΔ*	Hypoxic	0.94	0.12	3	n.s.
ERG2	*hap1AΔ*	Hypoxic	1.35	0.18	3	n.s.
ERG2	*hap1AΔ hap1BΔ*	Hypoxic	2.94	0.79	3	<0.01

Figure 10D - qRT-PCR Values

Gene	Strain	Condition	Mean RQ	St Dev	n	P-value
ERG5	2001 WT	Hypoxic	1.00	0.04	3	n.s.
ERG5	*hap1BΔ*	Hypoxic	1.04	0.06	3	n.s.
ERG5	*hap1AΔ*	Hypoxic	1.45	0.22	3	<0.05
ERG5	*hap1AΔ hap1BΔ*	Hypoxic	4.45	0.64	3	<0.0001

Supplemental Table S10 – Chromatin Immunoprecipitation Analysis (hypoxic conditions)

Figure 11A: ChIP qPCR Values

Gene	Promoter	Strain	Condition	Mean RQ	St Dev	n	P-value

ERG11	PDR	2001 WT	Hypoxic	1.11	0.65	3	n.s.
ERG11	PDR	*HAP1B-3XFLAG*	Hypoxic	0.92	0.52	3	n.s.

	**Figure 11B: ChIP qPCR Values**						
**Gene**	**Promoter**	**Strain**	**Condition**	**Mean RQ**	**St Dev**	**n**	**P-value**
ERG11	PPR	2001 WT	Hypoxic	1.04	0.36	3	n.s.
ERG11	PPR	*HAP1B-3XFLAG*	Hypoxic	3.68	0.61	3	<0.01

	**Figure 11C: ChIP qPCR Values**						
**Gene**	**Promoter**	**Strain**	**Condition**	**Mean RQ**	**St Dev**	**n**	**P-value**
ERG11	PDR	2001 WT	Hypoxic	1.09	0.42	3	n.s.
ERG11	PDR	*HAP1A-3XFLAG*	Hypoxic	4.86	1.75	3	<0.05

	**Figure 11D: ChIP qPCR Values**						
**Gene**	**Promoter**	**Strain**	**Condition**	**Mean RQ**	**St Dev**	**n**	**P-value**
ERG11	PPR	2001 WT	Hypoxic	0.80	0.19	3	n.s.
ERG11	PPR	*HAP1A-3XFLAG*	Hypoxic	0.41	0.10	3	<0.05

	**Figure 11E: ChIP qPCR Values**						
**Gene**	**Promoter**	**Strain**	**Condition**	**Mean RQ**	**St Dev**	**n**	**P-value**
ERG3	PDR	2001 WT	Hypoxic	0.85	0.14	3	n.s.
ERG3	PDR	*HAP1B-3XFLAG*	Hypoxic	0.77	0.19	3	n.s.

	**Figure 11F: ChIP qPCR Values**						
**Gene**	**Promoter**	**Strain**	**Condition**	**Mean RQ**	**St Dev**	**n**	**P-value**
ERG3	PPR	2001 WT	Hypoxic	1.00	0.09	3	n.s.
ERG3	PPR	*HAP1B-3XFLAG*	Hypoxic	3.09	1.03	3	<0.01

	**Figure 11G: ChIP qPCR Values**						
**Gene**	**Promoter**	**Strain**	**Condition**	**Mean RQ**	**St Dev**	**n**	**P-value**
ERG3	PDR	2001 WT	Hypoxic	0.90	0.14	3	n.s.
ERG3	PDR	*HAP1A-3XFLAG*	Hypoxic	1.99	0.21	3	<0.01

	**Figure 11H: ChIP qPCR Values**						
**Gene**	**Promoter**	**Strain**	**Condition**	**Mean RQ**	**St Dev**	**n**	**P-value**
ERG3	PPR	2001 WT	Hypoxic	1.01	0.17	3	n.s.
ERG3	PPR	*HAP1A-3XFLAG*	Hypoxic	19.33	6.03	3	<0.01

REFERENCES

1. Hickman MJ, Winston F. 2007. Heme Levels Switch the Function of Hap1 of Saccharomyces cerevisiae between Transcriptional Activator and Transcriptional Repressor. Molecular and Cellular Biology 27:7414-7424.
2. Zordan RE, Ren Y, Pan SJ, Rotondo G, De Las Peñas A, Iluore J, Cormack BP. 2013. Expression plasmids for use in Candida glabrata. G3 (Bethesda) 3:1675-86.
